# Venezuelan equine encephalitis virus infection causes chronic neurobehavioral outcomes, cellular remodeling, and hippocampal single-cell transcriptomic changes

**DOI:** 10.1371/journal.ppat.1014115

**Published:** 2026-04-08

**Authors:** Morgen VanderGiessen, Elizabeth Harris, Liduo Yin, Brittany Heath, Shannon K. Carney, Caitlin M. Woodson, Xiaowei Wu, Erik Johnson, Hehuang Xie, Michelle Theus, Kylene Kehn-Hall

**Affiliations:** 1 Department of Biomedical Sciences and Pathobiology, Virginia-Maryland College of Veterinary Medicine, Virginia Polytechnic Institute and State University, Blacksburg, Virginia, United States of America; 2 Center for Emerging, Zoonotic, and Arthropod-borne Pathogens, Virginia Polytechnic Institute and State University, Blacksburg, Virginia, United States of America; 3 Department of Statistics, Virginia Polytechnic Institute and State University, Blacksburg, Virginia, United States of America; 4 United States Army Medical Research Institute of Chemical Defense, Aberdeen Proving Ground, Maryland, United States of America; Washington University in Saint Louis, UNITED STATES OF AMERICA

## Abstract

Venezuelan equine encephalitis virus (VEEV), a neuroinvasive alphavirus, can cause significant neurological deficits in humans. Viral infections, including VEEV, have been linked to neurological diseases such as Parkinson’s and Alzheimer’s, though mechanisms remain unclear. Currently, not only are there no therapeutic options for VEEV available, but there is also limited information on the host responses following infection that contribute to neurological sequelae. To fill this gap in knowledge, longitudinal neuropathological, behavioral, and single-cell transcriptomic changes were examined in C57BL/6 mice intranasally infected with VEEV TC-83. Acute infection significantly altered inflammatory and innate immune single-cell signaling, induced astrocyte and microglia activation, and resulted in the loss of neurons in the hippocampus. Persistent motor dysfunction, memory impairment, and reduced anxiety-like behavior were observed up to 106 days post-infection (DPI) and more significantly in animals that displayed neurological symptoms during acute infection. These changes correlated with alterations in single-cell gene expression of synaptogenic signaling genes, neuron loss, and persistent glia cell activation at 106 DPI. Collectively, this study demonstrates that infection with VEEV induces chronic alterations in the hippocampus that correlate with neurological sequelae observed in human patients.

## Author summary

Viral infections may trigger persistent neurological conditions, such as chronic inflammation, that mimic or potentially induce the onset of age-related neurodegenerative disorders. In this study, we utilized Venezuelan equine encephalitis virus (VEEV), a mosquito-borne pathogen that can induce seizures and encephalitis, as a model to investigate the neurological consequences of viral infection. We identified long-lasting neurobehavioral alterations, including altered performance on elevated plus maze, novel object recognition, and modified-SHIRPA neuromuscular tests, predominantly in animals that presented with neurological symptoms. Immunofluorescence and single-cell RNA sequencing revealed persistent activation of microglia and astrocytes, along with neuronal loss in the hippocampus, a crucial region for learning and memory. Collectively, these findings highlight emerging evidence that viral pathogens can induce chronic neurological changes.

## Introduction

Viral infections may lead to persistent neurological symptoms that can mimic or trigger the onset of age-related neurodegenerative disorders. Previous reviews have explored the correlation between neurological disease and viral pathogens [1,2]. Moreover, emerging research suggests that certain neuroinvasive viruses, such as encephalitic alphaviruses, exacerbate neurodegeneration [3–6]. Research on other neuroinvasive arboviruses, particularly West Nile virus, found that infection can result in memory deficits and neurological disease that persist long after viral clearance [7,8]. These long-term impairments induce chronic microglia activation, synaptic remodeling, and neuronal loss, indicating that acute infection can drive long-lasting cognitive dysfunction. Therefore, characterization of the long-term neurological consequences of encephalitic alphavirus infection is critical, as these insights are necessary to inform the development of neuroprotective therapies and antivirals.

Alphaviruses are a large family of single-stranded positive-sense RNA viruses, belonging to the family *Togaviridae*, which can cause severe and potentially fatal neurological and arthritic disease following the bite of an infected mosquito. Venezuelan equine encephalitis virus (VEEV) infection in humans rarely results in death (<1%), but as high as 14% of cases progress to severe neurological disease which may result in the development of permanent neurological deficits consisting of but not limited to headache, seizures, sensory deficits, unconsciousness, motor dysfunction, and emotional changes [9–14]. Severe disease, neurological symptoms, and mortality is often observed in children, the elderly, and immunocompromised individuals [15–17]. While VEEV is primarily vector-transmitted, there is potential for its use as a bioterrorism agent, as it was studied as a bioweapon by both the United States and the former Soviet Union [18–20]. Despite its discovery in 1944 [21], there are still no well-characterized animal models that recapitulate chronic manifestations of disease, or publicly available vaccines or therapeutics licensed for human cases of VEEV.

Studies of VEEV in nonhuman primates and rodent models have identified overlapping neurological symptoms with humans, including photophobia, seizures, paralysis, rigidity, loss of consciousness, and behavioral alterations including anxiety and memory abnormalities [22–26]. Given substantial reports of memory dysfunction, and emotional alterations in human patients infected with VEEV, it is predicted the hippocampus, a portion of the brain vitally responsible for spatial navigation, emotional processing, learning, and memory, is substantially damaged following infection with VEEV [27]. Infection with other mosquito-transmitted viruses such as West Nile virus and Zika virus can result in learning and memory deficits, correlating with infection of the hippocampus, thus highlighting the important role of the hippocampus in viral neuropathogenesis [28–31]. Little is known about the impact of VEEV on hippocampal function in mice, but it has been shown that VEEV replicates within the hippocampus by 4 days post-infection (DPI) in a lethal mouse model [32,33].

A series of recent studies have investigated the neuropathological impact of alphaviruses on neuron loss and rodent behavior. Western equine encephalitis virus infection, often likened to a Parkinson’s-like disease, induced neuron loss, persistent astrocyte and microglia activation, and accumulation of alpha-synuclein in the substantia nigra pars compacta which correlated with motor gait abnormalities observed at 56 DPI [4,5]. Similarly, a combined neurodegeneration and VEEV infection model utilizing a transgenic mouse model of Alzheimer’s disease (Tg2576) suggested that VEEV infection worsens neuroinflammation and motor behavior [3]. Infection with VEEV has also been shown to increase the risk of developing amyloid beta plaques, which are a characteristic sign of Alzheimer’s disease [3,34]. The functional consequences of these neuropathological changes, especially those regarding memory dysfunction, have yet to be fully characterized. The exact mechanisms that induce neurological disease are also poorly understood, but likely involve a combination of various pathways including direct neuronal infection, disruption of the blood-brain barrier, and immune-mediated inflammation [32,35–39]. Furthermore, it has been well characterized that VEEV primarily infects neurons, but the nature of this infection and the long-term impacts on neuron function remains poorly understood.

To address these knowledge gaps, we utilized a sublethal model of VEEV infection to assess the neurological consequences of viral infection during acute and chronic timepoints. We used a sublethal dose of VEEV which elicited neurological symptoms and allowed for animal survival. We also evaluated memory, anxiety-like-behavior, and neuromuscular function of VEEV infected mice to characterize neurological consequences of viral infection. Finally, we characterized transcriptomic and neuropathological alterations within the hippocampus, a region of the brain associated with memory and anxiety, to determine hallmark features of neurodegeneration and restructuring that induce chronic neurological deficits. To this end, we performed single-cell RNA sequencing (scRNA-seq) coupled with virus-specific probes to evaluate differing gene expression between infected cells and bystander uninfected cells at acute and chronic time points post-infection. Moreover, we utilized this information to select cell populations to evaluate neuropathology via immunohistochemistry, to determine pathological features of VEEV infection, including neuron loss, microglia and astrocyte activation, and markers of neuronal activity which are chronically altered following infection with VEEV. Collectively, this study identifies a variety of long-term effects of viral infection on behavioral, transcriptomic, and neuropathological processes, which contribute to neurodegenerative phenotypes.

## Results

### VEEV TC-83 infection induces chronic weight loss, memory dysfunction, and reduced anxiety-like behavior in mice

**Fig 1:**
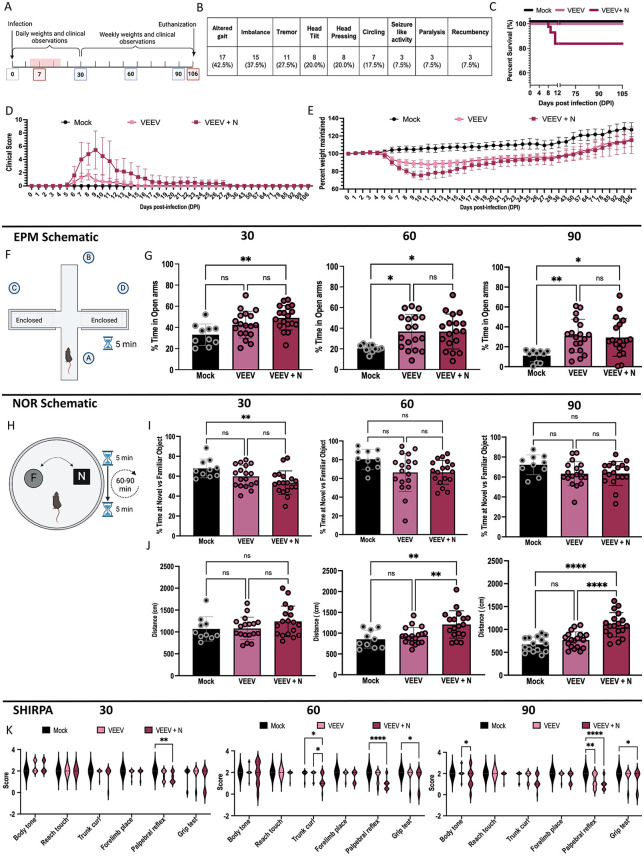
VEEV induces chronic weight loss, memory dysfunction, and reduced anxiety-like behavior in mice. **A)** Study timeline indicating collection timepoints (red) and behavioral assessments (blue). **B)** Neurological symptoms observed. **C)** Survival curves comparing mock-infected (Mock), VEEV-infected (VEEV), and VEEV-infected + neurological symptoms (VEEV + N). **D)** Clinical scores across groups. **E)** Percentage of weight maintained over time, relative to baseline weight. **F)** Schematic of the Elevated Plus Maze (EPM) test. **G)** Percentage of time spent in the open (unwalled) arms of the EPM at 30, 60, and 90 days post-infection (DPI). **H)** Schematic of the Novel Object Recognition (NOR) test. **I)** Percentage of time spent exploring the novel object versus the familiar object. **J)** Total distance traveled within the NOR arena. **K)** Results from SHIRPA neurological assessments. Data represents combined findings from two independent experiments: Mock (N = 10), VEEV (N = 18), and VEEV + N (N = 18). All Statistics shown are One-Way ANOVA with mean ± SEM error bars. The schematics in panels F and H were created in BioRender. Kehn-hall, K. (2026) https://BioRender.com/pb0mn1n.

To characterize a model of neurological consequences of viral infection, C57BL/6 mice were intranasally infected with 2 x 10^7^ plaque forming units (PFU) of VEEV TC-83. Mice labeled “Mock” are uninfected/control animals that have received intranasal PBS. A subset of mice was euthanized at 7 days post-infection (DPI; n = 10 VEEV; n = 10 mock/uninfected) to evaluate acute effects of infection and the remaining mice were followed to 106 DPI (n = 36–40 VEEV, n = 10 mock/uninfected) (Fig 1A). Mice were monitored and weighed at least daily for the first 4 weeks and then weekly until the end of the study. Mice that presented with neurological symptoms beyond 7 DPI are noted as VEEV + N and those without neurological symptoms are noted as VEEV. Mice were classified as VEEV + N if they had persisting (≥ two days) neurological symptoms (e.g., altered gait, imbalance, tremors, head tilt, head pressing, circling, seizure-like activity, partial paralysis, and recumbency). 100% of Mock infected animals survived (n = 10), 100% of VEEV infected (n = 18/18), and 81.81% of VEEV + N mice survived (n = 18/22) (Fig 1B). Neurological symptoms were observed primarily between 7 and 12 DPI; therefore, none of the 7 DPI animals were classified as having neurological symptoms. Mice infected with VEEV TC-83 lost significant weight and displayed clinical symptoms including changes in appearance, mobility, attitude, and body condition starting at day six post-infection (Fig 1D and 1E). Surviving mice slowly begin to regain weight and had little to no clinical symptoms by 15–16 DPI. Mice with neurological symptoms had increased clinical scores and weight loss as compared to non-neurological presenting VEEV-infected mice.

To assess the long-term impacts of VEEV infection, a series of behavioral and neuromuscular testing was performed at 30, 60, and 90 DPI. The elevated plus maze (EPM) is a standard assay of anxiety-like behavior in rodents [40,41] that can provide insight into the emotional changes observed following VEEV infection in humans [42]. Mice that spend more time in the enclosed (e.g., walled/closed) arms versus the open (e.g., unwalled/open) arms of the maze can indicate anxiety-like phenotypes (Fig 1F). In the EPM test, VEEV-infected mice showed reduced open arm dwelling time at 60 and 90 DPI compared to mock-infected controls (Fig 1G). A similar reduction in open-arm dwelling time was observed in VEEV + N mice at all timepoints (30, 60, and 90 DPI) further indicating alterations in behavior. The novel objection recognition (NOR) test assesses recognition memory, as memory deficits have previously been reported in humans infected with VEEV [42]. Prior to testing, naïve mice underwent preference testing to ensure no bias between the two objects (S1D Fig). For memory experiments, two identical objects (referred to as familiar objects) were placed in the arena, and mice were allowed to explore them for 5 minutes (Fig 1H). After a 60–90 minute retention interval, one familiar object and one novel object were placed in the arena, and the total time spent interacting with the objects were recorded. In this task, mice that spend greater time with the novel object is indicative of a potentially intact recognition memory. At 30 DPI, VEEV + N, but not VEEV mice, spent less time exploring the novel object compared to mock controls (Fig 1I). VEEV + N mice demonstrated increased distance traveled at 60 and 90 DPI compared to controls (Fig 1J), suggesting impairment of short-term memory and increased locomotion. To assess neuromuscular function, a modified and standardized version of the semiquantitative SHIRPA (SmithKline, Harwell, Imperial College, Royal London Hospital, Phenotype Assessment) testing, which has been previously used for to study VEEV infection in mice [43], was employed to evaluate the physical, behavioral, and neuromuscular phenotypes of mice following VEEV infection (Fig 1K). A score of 2 indicates normal responses, whereas values above or below 2 reflect hyperactivity or reduced performance, respectively. VEEV + N mice showed alterations in 1/6 of the parameters at 30 DPI, and 3/6 at 60 and 90 DPI. VEEV infected mice exhibited reduced responses in tests in 1/6 tests at 90DPI. Collectively, VEEV-infected mice exhibited impairments in visual-motor responses and limb strength consistent, but potentially less severe, than prior reports. Together, these results demonstrate that neuromuscular and behavioral performance are altered following VEEV infection, with mice that demonstrate neurological symptoms having decreased performance.

### VEEV infection significantly impacts single cell gene expression within the hippocampus at 7 DPI, including multiple immune and antiviral genes

**Fig 2:**
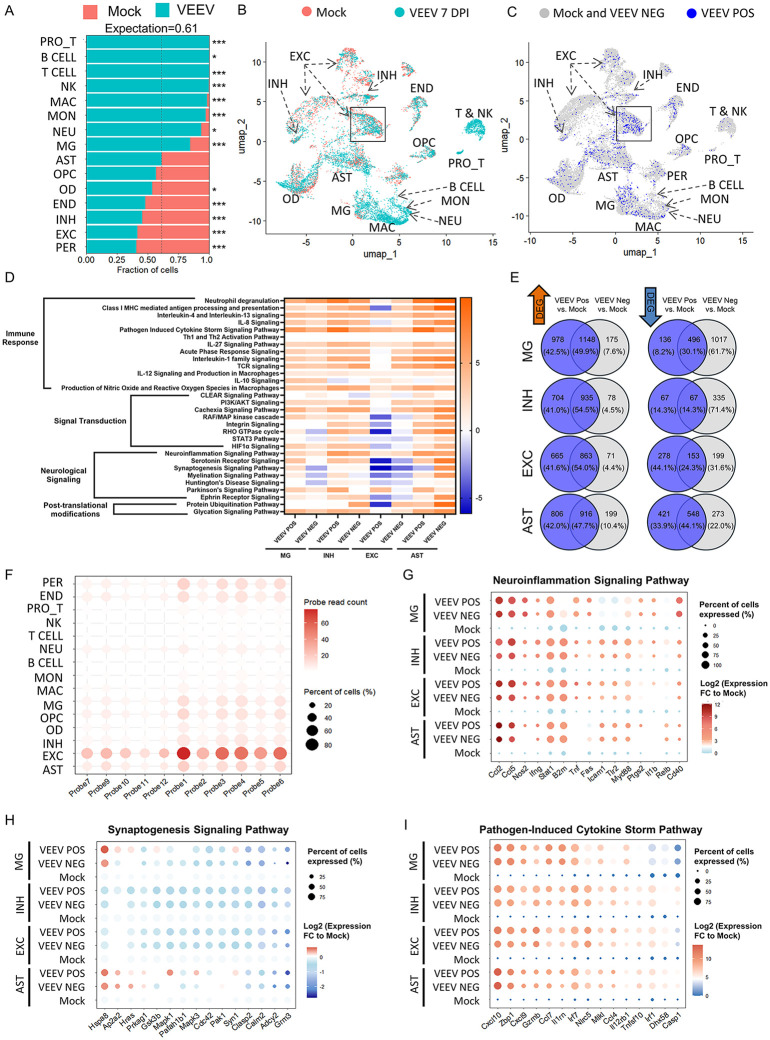
Single-cell RNA sequencing (scRNA-seq) analysis reveals transcriptomic alterations in the hippocampus at 7 DPI. **A)** Changes in cell abundance in response to viral infection. Statistical significance was determined by Fisher’s exact test,* = p-value≤0.05,*** = p-value ≤ 1e-10. **B)** Uniform Manifold Approximation and Projection (UMAP) plot showing differential annotated cell clusters in the hippocampus. **C)** UMAP identifying VEEV-infected cells detected via probe-based scRNA-seq. “VEEV NEG” represents viral probe negative cells sequenced from the VEEV 7 DPI dataset while “VEEV POS” represents cells which are probe positive from the same dataset. **D)** Top altered canonical pathways identified through Ingenuity Pathway Analysis (IPA). **E)** Venn diagram of differentially expressed genes of VEEV POS and VEEV NEG. **F)** Dot plot showing the expression of probes targeting VEEV viral RNA. Dot plot of genes from **G)** Neuroinflammation signaling pathway, **H)** Synaptogenesis signaling pathway, and **I)** Pathogen-induced cytokine storm pathway. EXC = excitatory neurons, INH = inhibitory neurons, MG = microglia, AST = astrocytes, PER=pericytes, OD = oligodendrocytes, OPC = oligodendrocyte progenitor cells, END = endothelial cells, MAC = macrophages, MON = monocytes, NEU = neutrophils, T = T cells, PRO_T = proliferating T cells, NK = natural killer cells.

To assess the impact of viral infection on critical cell populations related to memory and anxiety, we performed single-cell RNA sequencing (scRNA-seq) of pooled hippocampus from n = 5 animals for all groups. The number of sequenced cells was 4603 for mock mice and 7228 for VEEV 7 DPI mice. Cell populations critical for brain function were annotated using gene expression markers from published literature [44,45] for excitatory neurons (EXC), inhibitory neurons (INH), microglia (MG), astrocytes (AST), pericytes (PER), oligodendrocytes (OD), oligodendrocyte progenitor cells (OPC), endothelial cells (END), macrophages (MAC), monocytes (MON), neutrophils (NEU), B cells, T cells, proliferating T cells (PRO_T) and natural killer (NK) cells (S2A and S2B Fig). Total recovered cells were impacted by viral infection as indicated by cell type abundance (Fig 2A). Calculations were performed to assess the expected assortment of recovered cells as a result of viral infection using a traditional cell expectation calculation overlaid on the fractions of cells recovered in each group where the expectation = number of VEEV cells/ (number of mock cells + number of VEEV cells). VEEV infection substantially altered both the total number of cells (Fig 2A) and clustering diversity identified through Uniform Manifold Approximation and Projection (UMAP) plot showing differential annotated cell clusters in the hippocampus. The most striking differences were in the immune and microglia cell populations, with the majority of immune cells and microglia identified in VEEV-infected animals (p < 1e-3, Fisher’s Exact Test). Utilizing a UMAP to take multidimensional transcriptomic data and create a 2D clustering map, which presents more similar datapoints clustered together, and less similar datapoints to be further apart, we can highlight broad transcriptomic changes associated with VEEV infection. Cells recovered from VEEV-infected mice (Fig 2B, blue), displayed highly differential expression in several cell types, most notably immune cells and microglia as compared to mock (Fig 2B, red). Lymphoid immune cell clusters for both 7 DPI and 106 DPI were further analyzed and identified to be predominantly T-cells with few NK cells, proliferating T cells, and B cells (S3A- S3D Fig). Myeloid immune cell clusters were further analyzed and identified to be microglia, macrophages, monocytes and neutrophils (S3E-S3H Fig).

To elucidate the role of active VEEV infection in cells, rather than bystander effects from neighboring cells, we introduced a probe-based sequencing approach with a series of 11 probes specific for identifying different portions of the viral genome. Cells that were found to express all 11 of these probes, were recorded as VEEV positive (POS) whereas cells which do not express all 11 probes were recorded as VEEV negative (NEG). No VEEV POS cells were recovered in the mock mice, while 17% of cells in the VEEV-infected group were positive for all 11 probes (n = 1194/7228) and 83% did not contain all probes (n = 6034/7228). VEEV POS cells were most abundant in excitatory neurons (28%, n = 352/1259), while other cell types had between 1% and 21% infection rates (END 21% (n = 64/309); PER 21% (n = 35/170); AST 20% (n = 173/885); MG 19% (205/1067); OPC 18% (n = 43/238); INH 18% (n = 84/469); NEU 18% (5/28); OD 13% (n = 110/830); MAC 11% (82/719); MON 7% (17/260); B cells 6% (1/16); PRO_Ts 4% (6/148); T cells 2% (16/641); and NK 1% (2/191)) (Fig 2C). In addition, excitatory neurons had the highest average expression of all the viral probes (Fig 2C and 2F), indicating robust viral replication within this cell type. As expected, probes targeting the region of the genome encoding the structural proteins (Probe 1–6) had the highest average expression compared to probes targeting the nonstructural region (Probes 7, 9–12) (Fig 2F). These results confirm that neurons, specifically excitatory neurons, are the primary, but not only target, for active VEEV infection.

To elucidate the impacts of active viral infection state on gene expression, we investigated transcriptomic alterations using Ingenuity Pathway Analysis (IPA), a bioinformatics software effective for determining gene signatures associated with canonical pathways and upstream regulators [46–48]. Canonical pathway analysis, shown in a Z-score scale where orange indicates predicted activation and blue indicates predicted inhibition, identified activation of immune signaling in nearly all cell types, regardless of infection status (Fig 2D). VEEV POS EXC had the least predicted activation of immune signaling, which correlated with the highest level of viral probes detected. Signal transduction pathways including RAF/MAP kinase cascade and RHO GTPase signaling were inhibited in EXC, whereas the INH and AST displayed activation of the majority of the altered signal transduction pathways. Likewise, multiple neurological signaling pathways including Serotonin Receptor Signaling, Synaptogenesis Signaling, and Huntington’s Disease Signaling, were inhibited in EXC. MG had a mixed transcriptomic profile having predominantly activated pathways in both VEEV POS and NEG cells, but a few neurological signaling pathways were only inhibited in VEEV NEG cells. Neuroinflammation Signaling was activated in all cells types regardless of infection status. While the majority of the altered canonical pathways were similarly regulated between VEEV POS and VEEV NEG cells, there were some notable differences in DEGs between these cell populations. Greater than 45% of upregulated differential expressed genes (DEGs) were shared between the VEEV POS and VEEV NEG cells in EXC, INH, AST, and MG. In contrast, less than 31% of downregulated DEGs were shared in MG, INH and EXC, indicating that these cell populations have cell-type-specific responses in cells containing viral RNA (Fig 2E).

Among the most significantly altered canonical pathways were Neuroinflammation Signaling (Fig 2G), Synaptogenesis Signaling (Fig 2H), and Pathogen-Induced Cytokine Storm Signaling (Fig 2I). Differentially expressed genes within each pathway were compared across VEEV POS, VEEV NEG, and Mock samples. Across all cell types, key gene signatures, particularly those linked to cytokine production and inflammatory responses, including *Cxcl10, Ifn*γ, *Ccl2, Ccl5,* and *Stat1,* were consistently upregulated in the Neuroinflammation Signaling and Pathogen-Induced Cytokine Storm pathways (Fig 2G and 2I). These results are consistent with other published bulk RNAseq and scRNA-seq studies in models of VEEV infection [32,49], showing robust innate immune and antiviral gene responses during acute infection. *Stat1, Irf7, Ifnγ*, and *Tnf* were identified as significant upstream regulators via IPA. Both *Stat1* and *Irf7* were significantly upregulated in AST, MG, INH, and EXC in both VEEV POS and NEG cells (S4 Fig). In contrast, *Tnf* and *Ifnγ*, had cell-specific expression, with MG VEEV POS and NEG cells expressing *Tnf* and INH and EXC VEEV POS and NEG cells expressing *Ifnγ.* In contrast, genes within the Synaptogenesis Signaling pathway were predominantly downregulated, a trend that was consistent across both VEEV POS and VEEV NEG groups (Fig 2H), suggesting a virus-associated disruption of synaptic remodeling independent of direct viral detection. These findings indicate that VEEV infection broadly disrupts immune and synaptic signaling, marked by strong upregulation of inflammatory cytokine pathways and consistent downregulation of synaptogenesis-related genes in both probe-positive and probe-negative cells, highlighting a substantial bystander effect driven by infected cells.

### VEEV infection significantly impacts single cell gene expression within the hippocampus at 106 DPI, including the Glutaminergic Receptor Signaling Pathway

**Fig 3:**
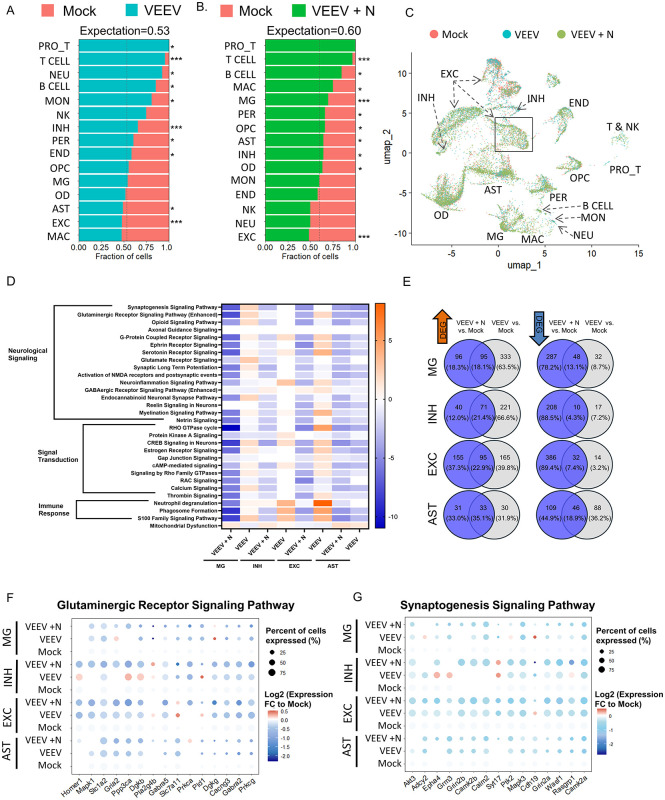
scRNA-seq analysis reveals transcriptomic alterations in the hippocampus at 106 DPI. **A-B)** Changes in cell abundance, between **A)** Mock vs VEEV and **B)** Mock vs VEEV + N. Statistical significance was determined by Fisher’s exact test,* = p-value≤0.05,*** = p-value ≤ 1e-10. **C)** UMAP plot showing differential annotated cell clusters in the hippocampus. **D)** Top altered canonical pathways identified through IPA. **E)** Venn diagram of differentially expressed genes of VEEV + N and VEEV. Dot plot of genes from **F)** Glutaminergic receptor signaling pathway and **G)** Synaptogenesis signaling pathway. EXC = excitatory neurons, INH = inhibitory neurons, MG = microglia, AST = astrocytes, PER=pericytes, OD = oligodendrocytes, OPC = oligodendrocyte progenitor cells, END = endothelial cells, MAC = macrophages, MON = monocytes, NEU = neutrophils, T = T cells, PRO_T = proliferating T cells, NK = natural killer cells.

To determine the long-term impacts of VEEV infection on gene expression in the hippocampus, scRNA-seq was performed on n = 5 pooled animals for all groups, Mock, VEEV, and VEEV + N, at 106 DPI. The number of sequenced cells was 3941 for mock, 4483 for VEEV, and 5839 for VEEV + N samples. Cell were again clustered into the cell populations (Figs 3C and S3) as described for the 7 DPI samples. VEEV probes were also included for these samples, but no probe-positive cells were detected, indicating that mice had cleared the infection within the hippocampus by 106 DPI. At 106 DPI, the total recovered cells were altered, with the most dramatic increase in immune cells in VEEV and VEEV + N mice (Fig 3A and 3B). The top altered canonical pathways across the cell types include pathways involved in neurological signaling, immune response regulation, and signal transduction (Fig 3D). Interestingly, the altered pathways at 106 DPI were largely predicted to be inhibited in AST based on their z-score as compared to 7 DPI where the majority of the pathways were active (compare Figs 2D and 3D). In contrast, altered pathways in MG, EXC, and INH had largely positive z-scores in VEEV mice, but predominately negative z-scores in VEEV + N mice, indicating differential regulation of these pathways between the two groups of mice. To compare upregulated and downregulated DEGs across EXC, INH, MG, and AST cell types, we analyzed the overlap between VEEV + N vs Mock (blue) and VEEV vs Mock (grey), with shared genes represented in the center (Fig 3E). At 106 DPI, fewer than 20% of downregulated DEGs were shared between the VEEV + N and VEEV groups. In contrast, a greater proportion of upregulated DEGs (ranging from 18% to 35%) were shared between the two groups, with the highest overlap observed in AST (35.1%) and the lowest in MG (18.1%). These results highlight differentially regulated genes present in VEEV-infected mice with neurological symptoms, particularly driven by alterations in excitatory neuron genes, and to a lesser extent, inhibitory neuron genes, pointing to a potential role for excitatory circuits in the manifestation of disease-related transcriptional changes.

To further characterize these genes, we identified substantial changes in the Glutaminergic Receptor Signaling Pathway (Fig 3F) and Synaptogenesis Signaling Pathway (Fig 3G), two highly related pathways essential for hippocampal function and neuronal activity because they underlie critical processes such as synaptic plasticity, learning, memory, and communication [50–52]. A large number of genes within these pathways were downregulated in all cell types including *Grin2b* (Fig 3G), a gene encoding a subunit of the NMDA glutamate receptor GluN2b*; Slc1a2*, the excitatory amino acid transporter 2 (*Eaat2*) protein-encoding gene, which is critical for removal of glutamate from neuronal synapses (Fig 3F) [53]; *Camk2a,* Calcium/Calmodulin Dependent Protein Kinase II Alpha, which regulates calcium signaling at the synapse [54] (Fig 3G); and *Mapk1*, mitogen activated protein kinase 3 (also known as *Erk1*; extracellular signal-regulated kinase) (Fig 3F). These changes correspond with VEEV + N mice having predicted inhibition of related downstream signaling pathways including cAMP and CREB signaling cascades [55]. In contrast, VEEV INH and EXC had predicted activation of these pathways, again highlighting transcriptional differences in these mice at 106 DPI. These data indicate that Glutaminergic Receptor Signaling and related downstream signaling pathways are suppressed in VEEV + N mice, correlating to behavioral alterations observed at 106 DPI.

### VEEV activates astrocytes and microglia in the hippocampus at 7 and 106 DPI

**Fig 4:**
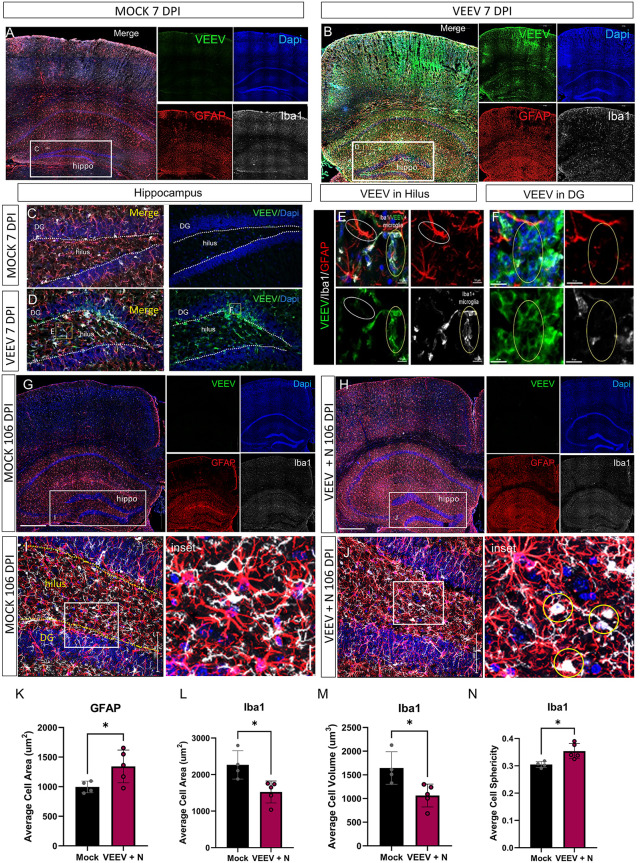
VEEV activates astrocytes and microglia in the hippocampus at 7 and 106 DPI. **A** and **B)** Wide-view images of the hippocampus (hippo) regions of the brain in mock (A) and VEEV infected (B) mice at 7 DPI. Sections were stained for VEEV infected cells (E2 glycoprotein, green), astrocytes (GFAP, red), and microglia/macrophages (Iba1, white), and a cell nuclei control (DAPI, blue). White boxes indicate regions further analyzed in subsequent panels. **C** and **D)** High magnification images of the hippocampus in C) mock-infected and D) VEEV infected animals. VEEV infected cells (E2 glycoprotein; green); Astrocytes (GFAP; red); Microglia/macrophages (Iba1; white). **E** and **F)** High magnification images of the hilus (E) and dentate gyrus (DG) (F) of the hippocampus demonstrating Iba1 + microglia/macrophages in an activated, amoeboid morphology in VEEV infected tissue and demonstrating increased activation of astrocytes in VEEV+ tissue through hypertrophied morphology. **G** and **H)** Representative wide-view confocal images of hippocampus (hippo) at 106 DPI with VEEV Envelope protein (VEEV E2; green), astrocyte (GFAP; red), microglia/macrophages (IBA1; white) expression in G) Mock and H) VEEV + N infected animals at 106 DPI. White boxes indicate areas investigated further in later panels for the hippocampus **(I and J)**. Astrocyte and microglia/macrophages in Mock infected animals have healthy, branched Iba1 + cells and fewer astrocytes in the hippocampus (I) whereas prolonged activation of astrocytes and Iba1 + cells, indicated by a circled regions contain activated ameboid microglia/macrophage structures, and more astrocyte coverage in the hippocampus are seen in VEEV + N 106 DPI samples (J). IMARIS quantification of **K)** GFAP average cell area, **L)** Iba1 average cell area, **M)** Iba1 average cell volume, and **N)** Iba1 average cell sphericity of VEEV + N 106 DPI vs Mock infected animals. n = 4 for Mock mice and n = 5 for VEEV + N mice. Statistical significance was determined by Student’s t- test,* = p-value<0.05.

To assess changes in microglia and astrocytes during acute VEEV infection, we conducted immunohistochemical analysis of key cell populations in the hippocampus, which are critical for the structure and function of this brain regions. To evaluate the localization of VEEV in the hippocampus, the envelope glycoprotein (E2), a marker of VEEV infection, was utilized to observe active infection. VEEV, shown in green, was found to readily infect the hippocampus at 7 DPI (Fig 4B), in agreement with other studies [32,56]. In mock infected mice, astrocytes (Glial fibrillary acidic protein (GFAP); red), and microglia/macrophages (Ionized calcium binding adaptor molecule 1 (Iba1); white) were found at a regular distribution with highly branched and ramified morphological structures, indicating a resting state overlapping with cellular nuclei (DAPI; blue) controls throughout the hippocampus (Fig 4A and 4C). A marker of astrogliosis, GFAP, shown in red, is increased in VEEV-infected mice at 7 DPI (Fig 4B). Morphological changes including the formation of ameboid-like structures indicating activated microglia, were observed in VEEV-infected mice, consistent with activation of the innate immune system and VEEV-induced neuroinflammation (Iba1; white) (Fig 4B). In the hippocampus, the hilar region and the dentate gyrus (DG) were examined in greater detail because of their critical functions. The hilar region plays a key role in regulating neuronal signaling pathways and maintaining network dynamics within the hippocampus, while the DG is essential for episodic memory formation, acting as a gateway for processing and encoding new information [57,58]. VEEV infection was localized in the hilus and the arc of the DG (Fig 4D). Formation of VEEV infection along the DG suggests this may be the site of entry of VEEV into the hilar region of the hippocampus. In mock infected mice, microglia and astrocytes were uniform across the DG and the hilus of the hippocampus (Fig 4C). Microscopic examination reveals microglia activation and increased colocalization around VEEV-infected cells, which may indicate the presence of either infected microglia or microglia engulfing infected cells (Fig 4E). In the hilus, VEEV infection was primarily found in the microglia, and substantial astrocyte activation was visible surrounding infected microglia (Fig 4E). In the DG, VEEV infection did not appear to overlap with microglia and astrocytes (Fig 4F).

To evaluate more chronic alterations in glia structure and activation states as a result of VEEV infection, we performed immunohistochemistry analysis of the hippocampus from 106 DPI groups. For these analyses, only mice that presented with persisting neurological symptoms (VEEV + N) were selected to be compared to mock mice. VEEV E2 was not detected in VEEV-infected mice at 106 DPI (Fig 4H), which is consistent with the lack of probe positive cells detected in the scRNA-seq studies. Consistent with the 7 DPI mock mice, microglia/macrophages in 106 DPI mock mice were relatively sparse with a healthy distribution and resting phenotype in both the wide view images (Fig 4G) and the zoomed-in representative images of the hippocampus (Fig 4I) at 106 DPI. In VEEV + N 106 DPI samples, an increase in the proportion of astrocytes and increased ameboid-activated microglia/macrophages were observed in the wide-view images (Fig 4H) and the zoomed-in portions of the hippocampus (Fig 4J). Morphology analysis was performed utilizing Interactive Microscopy Image Analysis Software (IMARIS) to assess the morphology of Iba1 and GFAP-expressing cells (Fig 4K–4N). In VEEV + N 106 DPI mice there was a significant increase in the average cell area of GFAP+ cells in the hippocampus compared to mock 106 DPI mice (Figs 4K and S5), which is consistent with hypertrophic activated astrocytes. Likewise, VEEV + N mice had a significant increase in both the average cell area and average cell volume of Iba1 + cells (Figs 4L, 4M and S5), which is consistent with an activated ameboid microglia shape. In further support of this, Iba1 + cells in VEEV + N mice had an increased average cell sphericity (Figs 4N and S5). Collectively, VEEV-infected mice showed acute and prolonged activation of microglia and astrocytes, consistent with previous reports in VEEV mouse models [3,32] and a persisting inflammatory response observed in multiple neurodegenerative diseases [59,60].

### VEEV infection results in hippocampal hilar interneuron loss

**Fig 5:**
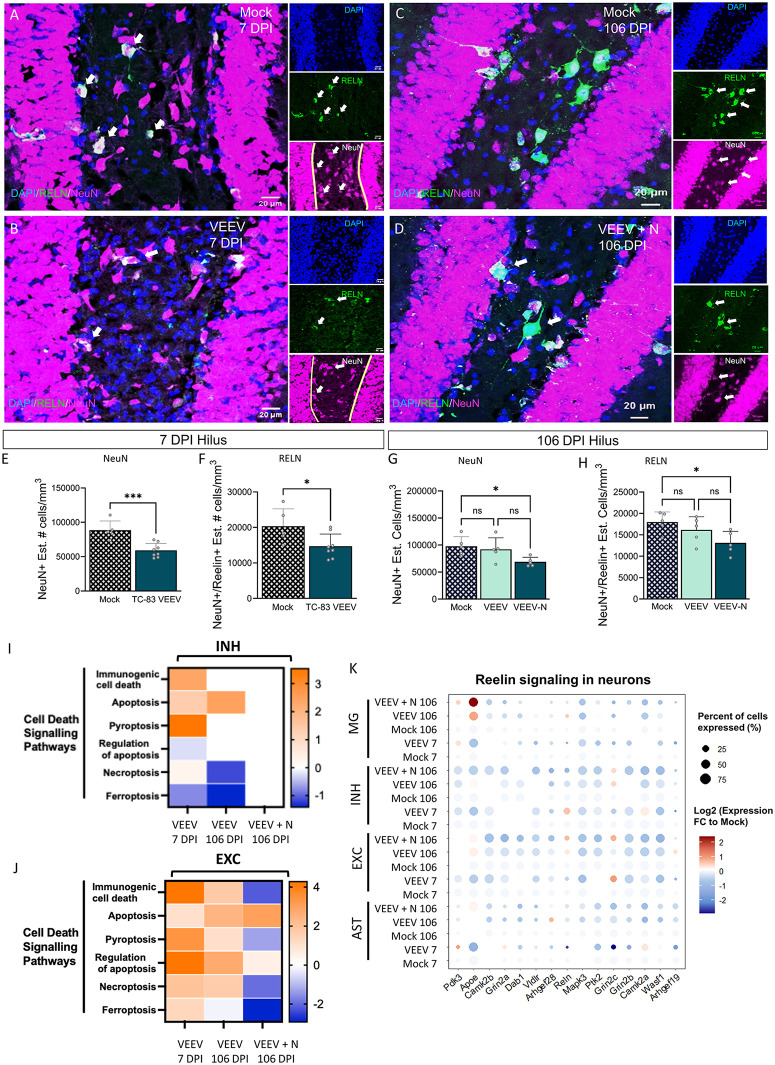
VEEV causes loss of neurons (NeuN) and inhibitory RELN-expressing neurons. **A)** Stereological quantification of NeuN (neuron nuclear marker; pink) and RELN (inhibitory neuron marker; green) in the hilus of the hippocampus at 7 DPI in **A)** Mock and **B)** VEEV and 106 DPI in **C)** Mock and **D)** VEEV. Quantified graph showing a significant decrease in **E)** NeuN+ cells and F) decrease in co-expressing of NeuN + RELN expressing cells in Mock vs. VEEV at 7 DPI. A loss of **G)** NeuN+ and **H)** NeuN + RELN expressing neurons persists to 106 DPI. **I)** Cell Death Pathways Identified via IPA of Neurons (INH and EXC). **K)** Dot plot of genes from Reelin signaling in neurons. All statistics performed with One-way ANOVA and Bonferroni post hoc correction; n = 5/group, ns = not significant, *p < 0.05; **p < 0.01, ***p < 0.001, ****p < 0.0001. Scale = 20µm. EXC = excitatory neurons, INH = inhibitory neurons, MG = microglia, AST = astrocytes.

To characterize neuronal changes in the hilus of the hippocampus, we quantified the total number of neurons using NeuN, a pan-neuronal marker, as well as Reelin (RELN) as a marker of interneurons (Fig 5A). The hilus of the hippocampus is predominantly composed of inhibitory interneurons [61,62]; therefore, an overall reduction in NeuN-positive cells (all neurons) is primarily indicative of interneuron loss in this specific region. VEEV-infected mice displayed a loss of interneurons in the hilus of the hippocampus at 7 DPI (Fig 5B and 5E), as indicated by reduced NeuN. This phenotype persisted through 106 DPI (Fig 5D and 5G). Reduced expression of Reelin-expressing interneurons was also observed at 7 DPI (Fig 5B) and 106 DPI (Fig 5D). Non-biased stereological quantification confirmed the loss of reelin-expressing neurons at 7 (Fig 5F) and 106 (Fig 5H) DPI. Transcriptomically, we observed an increase in apoptotic, pyroptotic, and immunogenic signaling at 7 DPI which may contribute to inhibitory neuron loss; however, at 106 DPI these cell death pathways are minorly observed in inhibitory neurons (Fig 5I) and downregulated in excitatory neurons (Fig 5J), which may be altered by the loss of inhibitory neurons, or excitatory neurons attempting to regulate signaling through inhibitory action (Fig 5I). Dot plot analysis of genes associated with Reelin signaling in neurons were analyzed across the 4 predominant cell types of interest, where a consistent downregulation was observed in nearly all cell types and conditions compared to mock. Reelin-pathway genes, including *Reln, Dab1, Camk2b* demonstrated negative fold changes supporting suppression of these genes. Additional neuronal signaling genes, including *Grin2a* and *Mapk3*, followed similar patterns, supporting a decrease in synaptic signaling as a whole including reelin-signaling pathways. By contrast*, Apoe* was both enriched in microglia and upregulated, supporting a disease-associated transcriptional response often associated with synaptic remodeling and injury response. Taken together, these results suggest that VEEV induced restructuring of the hippocampus as evidenced by loss of total hilar neurons (NeuN) and reelin-positive inhibitory neurons. Furthermore, these results are unexpected, as Reelin is traditionally associated with inhibitory interneurons, suggesting a shift in pathway activity toward excitatory neurons during infection.

### VEEV elevates c-FOS–mediated neuronal stress without affecting neurogenesis in the hippocampus

**Fig 6:**
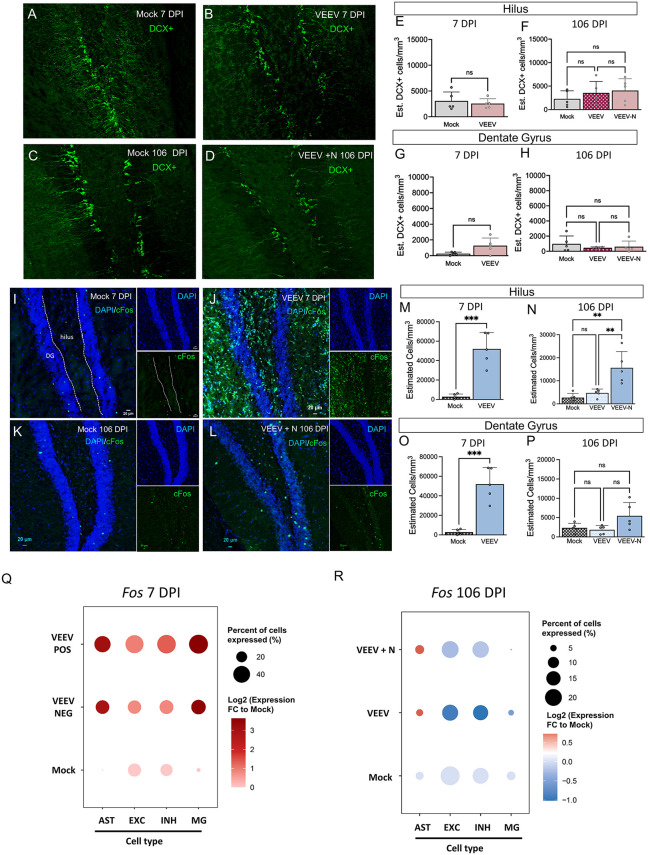
VEEV induces persistent neuronal stress (c-FOS) associated with memory loss in the hippocampus. **A-D)** High magnification images of DCX (green) in the hilus and DG of the hippocampus. **A)** Mock 7DPI compared to **B)** VEEV 7 DPI, quantified in the **E)** hilus and **G)** the DG of the hippocampus. **C)** Mock 106 DPI compared to **D)** VEEV + N 106 DPI, quantified in the **F)** hilus and **H)** the DG of the hippocampus. **I-L)** High magnification images of neuronal activity marker, c-Fos (green) expression in the hilus and DG of the hippocampus with DAPI (blue) as a cell nuclei control. c-Fos is lowly abundant in **I)** Mock 7DPI compared to **J)** VEEV 7 DPI, quantified in the **M)** hilus and **O)** the DG of the hippocampus. At 106 DPI **K)** Mock c-Fos expression is low compared to **L)** VEEV infected quantified in the **N)** hilus and **P)** DG. Analysis of *Fos* expression via scRNA-seq at **Q)** 7 DPI and **R)** 106 DPI. All statistics performed with One-way ANOVA and Bonferroni post hoc correction; n = 5/group, ns = not significant, *p < 0.05; **p < 0.01, ***p < 0.001, ****p < 0.0001. Scale = 20µm. EXC = excitatory neurons, INH = inhibitory neurons, MG = microglia, AST = astrocytes.

To assess whether VEEV disrupts neurogenesis and contributes to neuronal loss, we investigated doublecortin (DCX)-positive cells in the hippocampus across five groups: Mock 7 DPI (Fig 6A), VEEV 7 DPI (Fig 6B), Mock 106 DPI (Fig 6C), VEEV 106 DPI (S6C Fig), and VEEV + N at 106 DPI (Fig 6D), and found no significant changes in both the hilus (Fig 6E–5F) and DG (Fig 6G–6H), indicating that neurogenesis remains unaltered after viral infection. To assess whether loss of neurons in the hippocampus correlated with neuronal activity, we quantified c-Fos expression, an immediate early gene induced in response to neuronal activity, stress, and learning tasks [63–65]. An increase in c-Fos expression was observed at both 7 and 106 DPI in VEEV infected mice (Fig 6I–6L). Quantification showed that cFos was significantly elevated in the hilus and DG at 7 DPI (Fig 6M and 6O), but only within the hilus at 106 DPI (Fig 6N and 6P). We observed colocalization of c-Fos with neurons (NeuN) and astrocytes (GFAP) (S6G and S6H Fig). At 7 DPI, c-Fos colocalized with neurons (white arrows) and some c-FOS did not localize with neurons (yellow arrows), but may overlap with other cells such as activated astrocytes. At 106 DPI, c-Fos only colocalized with NeuN-expressing neurons (S6G and S6H Fig). Utilizing scRNA-seq data from the hippocampus, we observed an increase in c-Fos expression in VEEV POS and VEEV NEG astrocytes, microglia, inhibitory neurons, and excitatory neurons at 7 DPI (Fig 6Q). At 106 DPI, c-Fos expression was primarily observed in inhibitory and excitatory neurons, with mock and VEEV + N mice having similar expression levels (Fig 6R). The differences in c-Fos expression between the IHC and scRNA-seq data observed at 106 DPI could be reflective of differences in protein versus RNA levels in addition to the potential attempt of excitatory neurons to re-regulate c-Fos signaling post-infection. Collectively, these VEEV-induced alterations in c-Fos, most evident in the hilus and DG of the hippocampus, indicate a prolonged stressed state within the hippocampus and a potential reduction in synaptic function.

## Discussion

Previous reports indicate that VEEV infection in humans can lead to chronic neurological deficits, including seizure-like activity, memory loss, and emotional alterations, resembling age-related neurodegenerative diseases [3,24,33,66]. The hippocampus, a region of the brain associated with neurogenesis, memory formation, and emotional regulation, is particularly associated with these chronic deficits [67]. Here, we examined the impact of VEEV infection on hippocampal structure in animals with and without overt neurological symptoms. Hippocampal alterations were associated with changes in object recognition, neuromuscular function, and anxiety-related behaviors. Single-cell transcriptomic analysis revealed changes in key cell populations, including inhibitory and excitatory neurons, microglia, and astrocytes, consistent with the behavioral phenotypes observed. Collectively, the behavioral, structural, and transcriptomic changes observed in the hippocampus identified here help to establish a valuable animal model for interrogating the neurological consequences of VEEV infection and for guiding therapeutic strategies to mitigate chronic neuropathology.

Unlike previous fatal models of VEEV infection, our intranasal C57BL/6 model—using an immunocompetent mouse strain—recapitulates a gradient of disease severity with 10% mortality and 50% progression to neurological symptoms, more closely reflecting human infection (<1% mortality and <14% presenting neurological symptoms) than lethal models of VEEV infection [32,68,69]. Chronic manifestations of VEEV infection have been linked to neuromuscular deficits at 30 and 180 DPI, though Fongsaran et al., saw minimal changes in SHIRPA responses [3,33]. Here, we present modest neuromuscular function alteration, with significant changes primarily observed in the palpebral reflex, indicating potential alterations in visual-motor responses, and in the grip test, suggesting a reduction in neuromuscular strength, particularly in mice that manifest neurological symptoms. Taken collectively, variations in VEEV-induced neuromuscular function may depend on factors such as infection dosage, the presence or absence of neurological symptoms, external or environmental influences, and physiological state, such as animal stress.

Memory and anxiety-like deficits in alphavirus infection remain poorly understood. To assess these, we employed a limited set of behavior assays including a hippocampal-dependent working memory test, the NOR test [70], and a non-fear based amygdala-specific anxiety-like behavior assessment, through the EPM [71]. Substantial reduction of open-arm dwelling time in VEEV-infected mice was observed at 30, 60, and 90 DPI in mice that displayed visible neurological symptoms during acute infection indicating a reduction of anxiety-like behaviors. Novel object recognition was minimally altered at 30 DPI, while total exploration, measured by distance traveled, was altered at 60 and 90 DPI and may indicate aspects of locomotion or object interaction is chronically altered [72,73]. Other studies have shown that avoidance memory, a fear-based memory assessment useful for assessing hippocampal-related cognitive deficits, is substantially altered after VEEV infection, supporting our findings of damage to the hippocampus [74–76]. Overall, VEEV reduces anxiety-like behavior and recognition memory, though memory alterations may depend on activity levels and testing methods. Although these tests provide insight into hippocampal- and/or amygdala-associated function, each represents a single measure of a complex behavior process and could be strengthened through additional cohorts and additional behavioral tasks [77–79].

Several studies have investigated the role of acute transcriptomic alterations following VEEV infection, utilizing primarily bulk RNA-seq, microarray analysis, or quantitative polymerase chain reaction (PCR) [35,39,80–83]. ScRNA-seq on the hippocampus is notoriously challenging as neurons, critical for structure and function of the hippocampus, are easily damaged during cell dissociation leading to a loss of neuronal transcriptomes [84]. This study presents three novel research findings: 1) the first scRNA-seq analysis of neurons during VEEV infection; 2) the first transcriptomic analysis of chronic VEEV infection; and 3) the use of viral probes to elucidate the bystander effects of VEEV infection at acute time points. While this is the first application of viral probes in scRNA-seq for VEEV, prior studies have demonstrated the reliability of probe-based scRNA-seq for determining cellular infection states [85]. We used a stringent all or none criteria where detection of all 11 VEEV specific probes was required for a cell to be classified as VEEV-positive. While this approach eliminates false-positive designation of a cell as VEEV positive, a potential caveat is that cells designated as VEEV negative may have had a low level of virus replication occurring. The scRNA-seq analysis found a substantial proportion of hippocampal cells were infected with VEEV (17%) and of these infected cells, excitatory neurons had the highest expression of VEEV RNA within the hippocampus. The scRNA-seq data was driven by immune response signaling at 7 DPI, which may be reflective of such a large population of infected cells. However, we also observed significant induction of immune response genes in uninfected cells of VEEV infected mice, indicating that both direct viral infection and inflammatory responses contribute to pathogenesis during acute infection. Our 7 DPI scRNA-seq results are largely in agreement with other bulk transcriptomic studies which identified upregulation of genes associated with innate immune responses [32,35,39,80–83] and apoptosis [32,35,82]. At the time of this publication, only one other scRNA-seq study of VEEV infection *in vivo* has been published which focused on inflammatory gene expression of immune cell populations in an acute lethal mouse model of VEEV infection [49]. Our studies expand on this analysis to characterize alterations in neurons and astrocytes specifically within the hippocampus.

The upregulation of innate immune responses and inflammatory genes within astrocytes and microglia cells via scRNA-seq at 7 DPI suggests VEEV infection induced astrocytosis and gliosis. This was further supported by IHC analysis showing activation of microglia/macrophages and astrocytes at 7 DPI in the hippocampus. Glia cell activation was also observed at 106 DPI, even when viral RNA was not detected, indicating sustained activation of these cells in absence of active viral infection. This is consistent with prior observations of glial activation in VEEV-infected mice [3]. Specifically, prior work found that microglia/macrophage activation (Iba1) was increased globally across the hippocampus while astrocyte activation was found to be location-dependent with significant upregulation observed in the DG of the hippocampus, but not substantially altered in other regions of the brain such as the Cornus amonius (CA) regions 1 and 3 [3]. Our data further supports these findings by demonstrating activation of multiple immune response pathways at 106 DPI in microglia and astrocytes. Western equine encephalitis virus (WEEV), another encephalitic alphavirus which has previously been described as a Parkinson’s-like disease, has been shown to have persistent activation of astrocytes and microglia in mice [4,5]. Prolonged activation of astrocytes and microglia in VEEV + N mice was observed at 106 DPI and is consistent with an overall pro-inflammatory response state, which is commonly observed in neurodegenerative diseases [60,86].

Neurons in the hippocampus were found to be significantly impacted by VEEV infection at both acute and chronic time points. Notably, a loss in total neurons was observed at both 7 and 106 DPI which correlates with other studies showing the receptor for VEEV, LDLARD3 colocalizes with NeuN at 5 DPI [87], and that symptomatic animals have increased GFAP to NeuN ratios at 182 DPI [33]. Reelin (RELN), the protein product of the gene *Reln*, is an interneuron marker whose function mediates transient neuronal activity, is associated with synaptic plasticity, and is critical for neuronal migration [88]. RELN can also be expressed by GABAergic interneurons, pyramidal neurons, and interneurons critical for brain development, hippocampal morphogenesis, and supportive of memory formation [89–91]. In the adult cortex and hippocampus, reelin-expressing neurons are almost exclusively GABAergic [92,93]. RELN is a stable, extracellular protein critical for brain development and synaptic plasticity expressed in several regions of the brain, including the cortex and hippocampus [94,95]. Alterations in RELN are associated with multiple neurodegenerative diseases, including Alzheimer’s disease and schizophrenia [95]. Here, we observed a substantial loss in the number of RELN-expressing neurons in the hippocampus, a population of predominantly inhibitory GABAergic neurons, potentially due to acute upregulation of apoptotic, immunogenic, and pyroptotic cell death pathways. Multiple studies have shown that heterozygous reeler mice (HRM) mice, that have 50% reduction in the RELN protein, have behavioral abnormalities [96]. Importantly HRM mice display hippocampal-dependent learning deficits, specifically in contextual fear conditioned learning [97]. Given the reduction of RELN-expressing neurons following VEEV infection, we speculate that the reduction in inhibitory neurons alters the excitatory-inhibitory balance contributing to the observed behavioral deficits in these mice.

Chronic activation of neuronal activity was also observed, estimated by c-Fos expressing cells and supported by our scRNA-seq, following VEEV infection. Peripheral infection in other viral models of infection resulted in upregulation of c-Fos in the motor cortex, highlighting the importance of c-Fos as a potential biomarker of neuronal activity following viral infection [98]. Morphology associated with necrosis is observed in dopaminergic neurons, but neuron loss was not observed in the hippocampus despite substantial accumulation of alpha-synuclein, a hallmark of Parkinson’s disease, in WEEV-infected mice [4,5]. Activation of glutamate, the brains primary excitatory neurotransmitter, can increase expression of c-Fos, often observed in models of neurodegenerative diseases [99,100]. Unexpectedly, we observed cytoplasmic expression of c-Fos, rather than the typical nuclear localization, at 7 DPI. The cytoplasmic localization of c-Fos may be due to the ability of VEEV capsid protein to inhibit nuclear-cytoplasmic trafficking, as a means of dampening the innate immune response [101]. By 106 DPI, when VEEV antigen is no longer detectable, c-Fos is expressed in the neuronal nucleus, indicating a typical expression profile. However, scRNA-seq found that c-Fos expression was increased at 7 DPI but not 106 DPI in VEEV infected mice. A caveat of this analysis is that the scRNA-seq was performed with the entire hippocampus and the IHC was focused on the Hilus and DG. Increased expression of cFos in the hilus may indicate hyperactive interneurons or mossy cells, which can support regulation of DG granule excitatory neurons [102,103]. Our data suggests that the dysregulation of glutaminergic and dopaminergic signaling cascades lead to chronic activation of c-Fos which may contribute to neurological sequelae.

A limitation of our study is the use of VEEV TC-83, which is an attenuated strain of VEEV, that doesn’t cause disease in humans or equines. Infection of mice with wildtype strains of VEEV (e.g., Trinidad Donkey (TrD) or ZPC-378) results in uniform lethality, which limits the utility of these models for studying neurological sequelae [104]. However, there are some alternative approaches that could be explored in the future to study VEEV induced neurological sequelae using a wildtype strain of VEEV. Previous work with WEEV, which is also fatal in mice, has circumvented this issue through the use of immunotherapy to dampen viral replication, enabling animal survival while still inducing significant neurological disease [4]. Alternatively, neurological sequelae can be studied using other animal models such as non-human primates (NHPs). Cynomolgus macaques infected via the aerosol route with VEEV TrD or INH-9813 display clinical features that mirror human disease including fever, lethargy, and viremia as well as symptoms that suggest neurological involvement such as ataxia, tremors, hyperactivity, photophobia, and twitching [105–107]. NHPs recover from VEEV infection, but markers of neuroinflammation and brain injury were observed at 4-weeks post-infection [106]. While each of these approaches have their strengths and limitations, our findings as well as the findings of others [3,33] suggest that VEEV TC-83 infection of mice can be utilized as one animal model to study neurological sequelae.

In summary, this comprehensive study characterizes the neurological sequelae of VEEV TC-83 in immunocompetent mice, including pathological changes and transcriptomic alterations that may contribute to long-term memory and anxiety-like behaviors. Specifically, the data presented here detail the acute and chronic behavioral, transcriptomic, and neuropathological features in the hippocampus following VEEV infection, mirroring broader characteristics of neurological degeneration. Importantly, these studies present a clinically relevant mouse model of VEEV infection that results in severe CNS disease and neurological sequelae and can be utilized in future studies for evaluating neuroprotective and antiviral medical countermeasures.

## Materials and methods

### Ethics statement

All studies were reviewed and approved by both the Virginia Tech Institutional Animal Care and Use Committee (IACUC; #23–041) and Defense Threat Reduction Agency (DTRA) Animal Research Oversight Animal Care and Use Review Office (ACURO; CB11147.e001) before animal studies were started.

### Infection

Male 10–12-week-old wild-type C57BL/6 mice were ordered from The Jackson Laboratory and housed in an ABSL-2 laboratory at Virginia Tech. Mice were housed in groups of n = 5/cage and provided with food and water ad libitum. Standard 12 h light and 12 h dark cycles were used. Mice were anesthetized with vaporized isoflurane and then infected via intranasal inoculation with 2E7 PFU of VEEV TC-83 per mouse diluted in sterile phosphate buffered saline (PBS). Control mice were inoculated with PBS. Mice were monitored 1-2x daily for the first 28 days after infection in which appearance, mobility, attitude, and body condition on a scale of 0–3 or 0–4 per criteria (Table 1). Any mouse scoring the highest score in appearance, mobility and attitude were immediately euthanized. Attitude was scored as an observational index of the animals baseline responsiveness to being handled and lethargic, ranging from fully alert showing normal movement upon opening the cage and interacting with the animal (0), slowed or reduced exploration during handling, but aware [1], reduced responsiveness and alertness, minimal response to handling [2], and no observable reaction to handling, including static position and lack of movement [3]. Weight loss per se was not considered a feature requiring euthanasia unless weight loss exceeded 30% of the animal starting weight or was accompanied with additional severe illness or visual emaciation. Euthanized animals were counted as succumbing to viral infection when calculating mortality. Total score will determine whether additional monitoring or intervention will be given. A score of 0–5 will result in no change of monitoring (normal 1x daily), 6–10 will increase monitoring to 2x daily, and at ≥11 the animal is considered moribund and euthanized immediately. Additionally, any animal achieving the highest score in any category was immediately euthanized. Mice were also scored on a binary scale (0 = absent, 1 = present) for neurological symptoms including circling, head tilt, head pressing, altered gait, imbalance, and seizure activity as well as moribund symptoms including labored breathing, tremors, eye crust, lateral recumbency, and paralysis.

**Table 1:** Animal health and wellness score guide.

Appearance		Mobility		Attitude		Body Condition		Total Score	
0	Smooth coat, bright eyes	0	Active, exploring	0	Alert	0	Obese or Normal	0-5	*Normal 1x daily monitoring*
**1**	Slightly scruffy and/or hunched	**1**	Walking, less active	**1**	Mildly lethargic	**1**	Underconditioned	**6-10**	*Increased monitoring frequency - 2x daily*
**2**	Scruffy and/or hunched at rest	**2**	Slow movement	**2**	Lethargic	**2**	Emaciated	**≥11**	*Moribund, euthanize ASAP*
**3**	Very scruffy and/or hunched, mild eye crust	**3**	No Movement	**3**	Unaware			**E**	*Euthanized per study design*
**4**	Very scruffy and/or hunched, closed eyes	**4**	Unresponsive			**2** > 30% weight loss + additional clinical score (Alternative criteria)			
	*Any animal scoring the highest in these (greyed) criteria were euthanized*								

### Behavioral experiments

The elevated plus maze and novel object recognition tests were performed in a dark environment inside of secondary containment (Uline Rubbermaid cube truck; #H-8195) and cube truck lid; #H-8197). Tests were performed at approximately 30, 60, and 90 DPI in a consistent order (elevated plus maze, SHIRPA, novel object recognition) with one test being performed each day and at least one day off between tests. All mazes, tools, and surfaces were cleaned with 70% ethanol between each mouse and allowed to dry for at least 20 seconds between trials. A transport cage was cleaned between each cage of mice.

#### Elevated plus maze (EPM).

Elevated plus maze is a traditional test of animal anxiety-like behavior and mazes are publicly available (7001–0316, San Diego Instruments). Mice were placed in the center of the maze facing one of the open arms and recorded for 5 minutes. Time spent in each area (labeled as arm A, B, C, D and center) and total distance traveled was recorded and analyzed using animal TA software where mice were tracked using center body point mass (described in B.5.). Percent time spent in open (unwalled) was calculated by adding the proportion of time spent in the sum of the two open arms.

#### Modified SHIRPA.

SHIRPA testing of VEEV-infected mice has been previously described by other groups [3,66,108]. We performed a modified SHIRPA testing which included tests for body tone, reach touch, trunk curl, forelimb place, palpebral reflex, and grip test. Each test is briefly described below and score sheet for each test is displayed in (Table 2). Body tone test was performed by holding the mouse by the base of the tail on clean lab mat and gently depressing on the mouse’s spine using two fingers. Reach touch is performed by holding the mouse from the base of the tail approximately 6 inches high from the base of the biosafety cabinet and lowering the mouse towards an empty cage until they either touch the base of the cage or extend forelimbs and reach towards the cage. Trunk curl is performed by holding the mouse from the base of the tail at least 6 inches above the base of the biosafety cabinet and assessing the mouse ability to perform a trunk curl. For this test, mice were provided three attempts to exceed 90-degree trunk curl (if necessary) and provided 30 seconds between each test to rest. Palpebral reflex was performed by holding the base of a mouse tail and approaching it from 3 directions, in front of the animal (between the eyes), and from the left, and right sides of the animal towards the sides of the skull. Animals were approached until a response could be recorded (blinking, movement, etc.) or until the q-tip is approximately 2 mm from the animal to ensure they are not contacted by the q-tip. The grip test was performed by allowing a mouse to grab a metal grate and then inverting them at 10 inches above the base of the biosafety cabinet and recording the grip duration up to 60 seconds. For this test, mice were provided three attempts to exceed 60 seconds (if necessary) and provided 30 seconds between each test to rest.

**Table 2:** Modified SmithKline, Harwell, Imperial College, Royal London Hospital, Phenotype Assessment (SHIRPA) score guide for neuromuscular function assessment.

Test	0	1	2	3
Body Tone	No response, no sensation	Physical depression of spine	Resistance, slight response to touch	Hunches, Leaches away from touch, aggression
Reach Touch	No actions, No reaching, clasping of hindlimbs	Delayed reach, reach after whisker touch to surface	Promptly reaches toward touchable surface	Hyperaggressive, swinging and/or biting
Trunk curl	No response, clasping of hindlimbs	Curls <90°	Curls ≥90°	Climbs up tail, swinging and/or biting
Forelimb place	Leg does not move, stays where placed	Moves, but does not return to original position	Returns position quickly	Hyperactive or aggressive response, biting tools
Palpebral reflex	No response	Slow blink, blink on one side, not other	Quick Blink	Repetitive blinking after stimulus removal
Grip Test	Does not grip/ Noncompliant	Fail: Grips <60 seconds	Pass: Grips ≥ 60 seconds	N/A

#### Novel object recognition (NOR).

Novel object recognition is a common recognition memory test used for rodents. For these experiments, a rounded bucket with a 10-inch internal diameter was purchased (2-gallon bucket, Item # 1092675, Manufacturer # 1123008, Ace Hardware). Markings were made on the interior of the maze bucket to distinguish different sides. On day 1, habituation was performed mice were placed into the maze and allowed to explore for 5 minutes and then removed. On day 2, familiarization was performed by placing a mouse into the maze with two familiar objects (round silver cabinet door knobs, Model # BP3710226; SKU: 1009255781 Home Depot) and allowed to explore for 5 minutes then removed. Each object had either a 1-inch diameter (round), or 1-inch length (square) placed 2 inches apart. After 60–90 minutes mice were reintroduced into the maze with one familiar object and one novel object (black square cabinet doorknobs Model # P41758C-FB-C, SKU: # 1008539424, Home Depot) and again allowed to explore for 5 minutes. Percent time at novel object was calculated by (time at novel object/(time at novel object+ time at the familiar object)) *100.

#### Animal TA.

Animal TA is a free tracking software performed for all maze analyses. Each set of mp4 videos were uploaded and converted into AVI files. Each video was cropped to ensure that video durations are within +/-2 seconds length of all videos in the batch (~300 seconds/5 minutes of analysis per video). Software performs background corrections to ensure that only the single animal in the maze is tracked, and a reference length was added to allow conversion of pixel distances, to real distance from the center of the maze to the outer leg of the arm (NYM 38.1 cm, EPM 36 cm) or the diameter of the maze (NOR, 26 cm). Each mouse was tracked from the center body mass where entry of the animal into an arm of a maze takes place when the center mass crosses the arm threshold from the center point.

### IHC Perfusion fixation and organ dissection

Mice were euthanized via CO_2_ and blood was collected via cardiac puncture. Dissection was performed to expose the heart, and then 20 mL heparin diluted in ice-cold sterile PBS (2000 units/kg) was injected via the apex of the heart into the left ventricle. A small incision in the right atrium was made, and 50 mL of ice-cold 4% paraformaldehyde and 3% sucrose in PBS solution (pH = 7.4) was slowly injected into the same puncture site at a rate of 5 mL/minute to flush blood from the circulatory tract. Brains were dissected from the mice and placed in the 4% paraformaldehyde and 3% sucrose in PBS solution for 4 hours on ice. Brains were then washed with PBS for 5 minutes (~20mL) and then preserved in a sucrose gradient until brains sunk to the bottom of a 50 mL conical tube (20% for ~48h; 25% ~ 48h, all at 4°C) and then molded in OCT solution (30% OCT + 1:1 ratio of 60% sucrose and PBS) and stored at -80°C before cryosectioning.

### Brain molding, sectioning, and slide preparation

Following sucrose gradients, brains were molded and frozen in a solution containing 30% OCT and 60% sucrose dissolved in PBS and slow frozen for 30 minutes on dry ice before storage at -80°C for sectioning. The molds were filled at least 2mm beyond the brain in the molds with OCT (Tissue-Plus O.C.T. Compound, # 23-730-571, Fisher Scientific Waltham, MA, USA) and slow frozen for 30 minutes on dry ice. Brains were then placed into the –80°C for storage until cryosectioning. The brains were trimmed through the coronal plane of the brain through the center of the cerebellum which was then mounted on a microtome. Cryosectioning was performed via a cryostat (CryoStar NX50, #957130, Thermo Scientific, Waltham, MA, USA) from -1.1 to -2.6 mm posterior from bregma at -20° and 30μM coronal sections were obtained with 5 sections per slide, spaced 450μM apart. Sections were dried to adhere to the slide at 40°C for at least 1 hour and subsequently stored at -80°C for staining.

### Immunohistochemistry analysis

Immunohistochemistry analysis was performed to assess Microglia (Iba1), Neurons (NeuN), inhibitory neurons (RELN), Astrocytes (GFAP), Neural activity (c-FOS), and viral presence (VEEV E2) in the hippocampus using the antibodies and dilutions shown in Table 3. Wide-view images of the hippocampus were taken on a Zeiss LSM 880 (ZEISS, Oberkochen, Germany) with a Zeiss FLUAR 5x/0.25 lens (#440125, ZEISS, Oberkochen, Germany) and Zeiss 63X LD C-Apochromat 63x/1.15 water emersion lens (#421887, ZEISS, Oberkochen, Germany).

**Table 3:** Antibodies used for IHC.

Antibody	Concentration	Product Number	Supplier
Iba1 (Rt*)	1:250	ab283346	Abcam, Waltham, MA, USA
GFAP (Rb**)	1:250	12389f	Cell Signaling Technology, Chantilly, VA, USA
NeuN (Rb) NeuN (Rt)	1:500 1:500	12943S/ ab279297	Cell Signaling Technology, Chantilly, VA, USA Abcam, Cambridge, MA, USA
Reln (Gt***)	1:50	AF3820	R&D Systems, Minneapolis, MN, USA
c-FOS (Rb)	1:100	2250	Cell Signaling Technology, Chantilly, VA, USA
VEEV E2 clone 1A3B-7 (Ms****)	1:100	MAB8755	Sigma, St. Louis, MO, USA
DAPI Fluoromount-G	N/A	0100-20	SouthernBiotech, Birmingham, AL, USA
Alexa Fluor 647 Donkey anti-rabbit	1:250	A31573	Invitrogen, Carlsbad, CA, USA
Alexa Fluor 594 Donkey anti-rabbit	1:500	A21207	Invitrogen, Carlsbad, CA, USA
Alexa Fluor 488 Donkey anti-rabbit	1:250	A21206	Invitrogen, Carlsbad, CA, USA
Alexa Fluor 488 Goat anti-Mouse	1:250	A21202	Invitrogen, Carlsbad, CA, USA
Alexa Fluor 647 Donkey anti-rat	1:250	A48272	Invitrogen, Carlsbad, CA, USA
Alexa Fluor 488 Donkey anti-Goat	1:250	A11055	Invitrogen, Carlsbad, CA, USA
Alexa Fluor 594 Donkey anti-Goat	1:500	A2185074	Invitrogen, Carlsbad, CA, USA

*Rt=rat; **Rb=rabbit; ***Gt=goat; ****Ms=mouse.

Quantitative analyses and representative images of the dentate gyrus and hilar regions of the hippocampus were collected by a blinded investigator. Five coronal sections were observed using the Optical Fractionator probe in Stereo Investigator software (MBF Bioscience, version 2017.03, Williston, VT, USA) with an Olympus BX51TRF motorized microscope (Olympus America, Center Valley, PA, USA), following previously established protocols [109–113]. Regions of interest, including the DG and hilus, were outlined, and grid dimensions for the optical fractionator were set to 150 × 150 µm with a 75 × 75 µm counting frame. The estimated cell count was normalized to the contoured volume using planimetry, providing the number of cells per mm³.

### IMARIS analysis

Three-dimensional image analysis was performed using the z-stack confocal images and IMARIS software version 9.8.2 (Oxford Instruments, Concord, MA, USA). For all analyses, surfaces were generated with consistent parameters across images, using a smoothing factor of 0.625 and a background subtraction (local contrast) defined by the diameter of the largest sphere that fits into the object (2.58 µm). For individual cell morphology analyses, cells were rendered using the surface creation wizard with split touching object enabled to separate adjacent cells with a minimum object separation of 6.5 µm. Only well-rendered cells fully contained within the hilus were included for analysis; incomplete or poorly rendered cells were excluded following visual inspection. Three brain sections were analyzed per mouse with 4–17 well-rendered cells being retained per image for morphology analysis for both Iba1-labeled microglia and GFAP-labeled astrocytes. Cell volume, area, and sphericity were extracted for Iba+ cells and cell area for GFAP+ cells. N = 5 per group were analyzed. However, one Mock mouse had substandard tissue quality resulting in suboptimal staining and was excluded from the final analysis, resulting in N = 4 for Mock and N = 5 for VEEV + N.

### scRNA-seq sample collection

Mice were euthanized via CO_2_ asphyxiation and secondarily euthanized via cardiac exsanguination. Mice were perfused with 10mL heparin (2000 units/kg) or 1% EDTA in phosphate buffered saline (PBS) via the apex of the heart into the left ventricle followed by 50mL of PBS. Brains were dissected and left hemisphere was placed into 10% formalin. From the right hemisphere, the hippocampus was dissected and placed into 10x genomics fixation buffer solution (Chromium Next GEM Single Cell Fixed RNA Sample Preparation Kit #1000414, 10x Genomics, Pleasanton, CA). N = 5 pooled hippocampus was minced in a petri dish until they could be pipetted through a wide bore pipette tip and weighed to achieve 30mg total fixed tissue. After 24-hour fixation, the pieces were spun down and washed with PBS following 10X sample fixation protocol and stored at 4°C prior to sending samples out for sequencing.

### scRNA-seq sample purification, processing, and analysis

scRNA-sequencing was performed by MedGenome (Foster City, CA). Fixed and quenched tissues were dissociated for 10 minutes at 37°C using an Miltenyi Octo Dissociator (Mitenyi Biotec, Gladbach, Germany #130–096). Dissociated tissue were filtered through a 30μm filter to remove debris and undissociated tissue pieces, and centrifuged at 850 rcf for 5 minutes. The cell pellet was resuspended in chilled quenching buffer. Cells were stained with ethidium homodimer-1 and counted using countess II FL. The cell concentration was determined based on the reactive fluorescent protein positive concentration. For custom probe spike in, single cell gene expression flex probe configuration was used with sequences (Table 4) ordered from Integrated DNA Technologies (Integrated DNA Technologies, Coralville, IA). Each probe represents 25 bp sequences homologous to the target VEEV viral RNA (VEEV genome identified from NCBI GenBank: L01443.1). Pool 1 (Probe pairs 1–6) target the VEEV structural polyprotein gene and Pool 2 (Probe pairs 7–12) target the VEEV nonstructural polyprotein gene. Note that Probe 8 resulted in no captured sequences from any sample and therefore this probe was excluded from the data analysis. One – two million cells were hybridized with a modified probe hybridization mix containing mouse WTA probes BC001 and custom probes, each probe at 40 nM for 16–24 h at 42°C. Each Left-hand probe (LHS) contained a linker region 5’-CCTTGGCACCCGAGAATTCCA-target_LHS-3’ and right-hand probe/5Phos/-target_RHS-ACGCGGTTAGCACGTANNACTTTAGGCGGTCCTAGCAA-3’. Following probe hybridization, gel beads in emulsions were generated for 6000 cells targeted recovery using 10x genomics chromium fixed RNA profiling reagent kit (10X Genomics, Pleasanton, CA, #1000414). The libraries were generated and sequenced in NovaSeq X for 120M paired reads.

**Table 4:** Probe Identifiers for scRNA-seq. All sequences listed 5’- > 3’.

Pool ID	Probe ID	Left Hand Probe	Right Hand Probe
1	Probe Pair 1	ACATGACGACTGAAAGGGCTGTCCT	AGATCCTTCATTCACACCTCCCAGC
1	Probe Pair 2	AGTCTAACATAACCGTCGTGCCCGT	CGCTCTTTACTGCCTCGATTGCTAT
1	Probe Pair 3	ACCTGAGCAGGCGAGTCACTACGAT	CAAGGCGGCCAGAGGGATCAGCAAT
1	Probe Pair 4	GTGTCGGTGTTTCTGACACCCTGGT	GAACAAGGCGTCGGGAATGTCAAAG
1	Probe Pair 5	TCTGCAAAACAGCCAGGTAGACGCT	GCAACGGAAACGGTTGCAATGGCGG
1	Probe Pair 6	GTAAGTTGGAGTGCATTCCTGAGAT	CCGCAGCATTTGATGGCTGGTGAAT
2	Probe Pair 7	CGCCGAATCCAATACGGGCAATTCT	CTCATTTGCGTGACATTGCAAT
2	Probe Pair 8	GAATGTTCGGAAGCAGGACCGCATT	AGATCCTTCATTCACACCTCCCAGC
2	Probe Pair 9	AAAGGAACAGGATGCAGGGTTCGGT	AGCACTCCACTTTTCCTTCTGCCTT
2	Probe Pair 10	ACTCTTCGACGGGGCATTTCGACCT	AATACTGCTCATGCTTTCTCCGAG
2	Probe Pair 11	TTCAGCGAGAGGGTGGATGCAAGAT	AATTTTTCACTCTTGAGTACAGCCT
2	Probe Pair 12	TATTGCATGTCCCTCTGGCACCACT	ACTTTACCATGGTATGGTTCCACGG

The raw sequencing reads were trimmed and aligned to the mouse reference genome (mm10) using Cell Ranger (10x genomics, v.8.0.1) and quantified using ‘Cell Ranger count’. The resulting count matrix was further processed using the R package Seurat (v.5.1.0) for quality control and downstream analysis [114]. The following filtering steps were applied to remove low-quality cells, cells with the number of expressed genes (denoted as nFeature_RNA) over 200 and cellular mitochondrial genes detected < 10% were retained. Based on the 2,000 most variable genes, the normalized scRNA-seq count matrix was used for subsequent PCA analysis. The first 20 PCs were used for uniform manifold approximation and projection (UMAP) and the shared nearest neighbor (SNN) computation. The harmony package (v1.2.1) was adopted to remove batch effects among samples [115]. The gene expression comparison between samples was performed by FindMarkers function in Seurat package based on a Wilcoxon Rank Sum test with parameter “min.pct=0.05”. The non-parametric statistical method, Wilcoxon test compares the ranks of gene expression values between two groups without assuming normal distribution, making it robust for sparse scRNA-seq data. It tests whether the expression distributions are stochastically equal, with significant p-values indicating differential expression. Gene expression violin plots were generated, with asterisks indicating statistical significance levels as: *p < 0.05, **p < 0.01, and *p < 0.001 (ns = not significant, p ≥ 0.05). Cell type markers were identified utilizing published literature as shown in Table 5.

**Table 5:** Markers used for cell annotation.

Cell type	Abbreviation	Markers	Reference
Astrocytes	AST	Aqp4, Slc1a3	[44]
Excitatory neurons	EXC	Slc17a7, Slc17a6	[116]
Inhibitory neurons	INH	Gad1, Gad2	[44]
Oligodendrocytes	OD	Mog, Mag, Ermn	[44]
Oligodendrocyte precursor cells	OPC	Cspg4, Vcan	[44]
Neutrophils	NEU	S100a8, S100a9	[117]
Microglia	MG	Tmem119, Trem2, P2ry12	[118]
Macrophages	MAC	Gpnmb, Spp1	[117]
Monocytes	MON	H2-Aa, H2-Ab1, H2-Eb1	[117]
T cells	T CELL	Trac, Cd3e, Cd3d	[119]
B cells	B CELL	Cd79a, Cd79b	[117]
Natural Killer cells	NK	Klre1, Ncr1, Cd244a	[119]
Proliferating_T cells	PRO_T	Tyms, Rrm2, Top2q	[119]
Pericytes	PER	Dcn, Vtn	[120]
Endothelial cells	END	Pecam1, Cldn5	[121]

### Ingenuity pathway analysis

Filtering was performed to P-value cutoff p < 0.0001. Z-scores that are not a number (pathway activation or inhibition are unable to be predicted) are displayed as zero on the heatmaps.

### Statistical analysis

Statistical analysis for the scRNA-seq data is described above. For all other data, unless otherwise specified, statistical analyses involving two or more groups were conducted using a one-way ANOVA, with asterisks denoting significance levels as follows: p > 0.05, *p < 0.05, **p < 0.01, ***p < 0.001, ****p < 0.0001. For comparisons between two experimental groups, a Student’s two-tailed t-test was applied. All statistical analyses were performed using GraphPad Prism Version 10.

## Supporting information

S1 Fig Animal methods and validations. A) percent weight maintained, B) Raw weight (g), and C) Clinical score of 7 DPI cohort Mock vs VEEV-infected animals. D) Percent time at object 1 vs object 2 in naïve untrained mice.
(TIF)

S2 Fig scRNA-seq cell type annotation. A) Heatmap map showing the expression level of selected cell type markers for each cluster. B) Dot plot showing the expression of select cell-type markers.
(TIF)

S3 Fig Immune cell clustering analysis. A) Total cell clusters identified as lymphoid immune cell clusters. B) Cell clusters containing B cells (red); Natural Killer (NK; green), Proliferating T-cells (blue), and T-cells (purple). C and D) lymphoid immune clusters colored by sample. Separated immune cell clusters for C) 7 DPI and D) 106 DPI. E) Total cell clusters identified as myeloid immune cell clusters. F) Cell clusters containing Macrophage (red); Microglia (green), Monocyte (blue), and Neutrophils (purple). G and H) Myeloid immune clusters colored by sample. Separated immune cell clusters for G) 7 DPI and H) 106 DPI.
(TIF)

S4 Fig Violin plots of upstream regulators identified via Ingenuity Pathway Analysis (IPA) of scRNA-seq data from Mock vs VEEV 7 DPI samples. A) *Ifnγ*, B) *Irf7*, C) *Stat1*, and D) *Tnf*. Statistical significance was determined by Fisher’s exact test,* = p-value≤0.05,*** = p-value≤0.001,**** = p-value≤0.0001.
(TIF)

S5 Fig Representative images of GFAP+ and Iba+ cell morphology analysis. (A-B) GFAP+ cell 3D reconstruction in the hippocampus of Mock 106 DPI and VEEV + N 106 DPI mice, respectively. (C-D) Bottom panels show inset which reveals larger more hypertrophic astrocytes in VEEV + N mice. (E-F) Iba1 + cell 3D reconstruction in the hippocampus of Mock 106 DPI and VEEV + N 106 DPI mice, respectively. (F-H) Bottom panels show inset which reveals VEEV + N mice have a morphological shift in microglia morphology to ameboid shape.
(TIF)

S6 Fig Representative Images for neural progenitor and neural activity. Doublecortin (DCX+) cells in A) Mock, B) VEEV, and C) VEEV + N at 106 DPI. cFos+ cells in A) Mock, B) VEEV, and C) VEEV + N at 106 DPI. G) Representative images of c-Fos expression overlayed with astrocytes (GFAP), and neurons (NeuN) in VEEV-infected animals at 7 DPI indicate partial overlap with neurons (NeuN; pink) (overlap indicated by white arrows, non-overlap indicated by yellow arrows) compared to astrocytes (GFAP; red) at 7 DPI, whereas H) 106 DPI displays c-Fos almost exclusively in neurons (white arrows).
(TIF)
