# Supplementary figures and images for: Venezuelan equine encephalitis virus infection causes chronic neurobehavioral outcomes, cellular remodeling, and hippocampal single-cell transcriptomic changes
Source: PLoS Pathog. 2026 Apr 8;22(4):e1014115. doi: 10.1371/journal.ppat.1014115 (PMC13089902; doi:10.1371/journal.ppat.1014115)

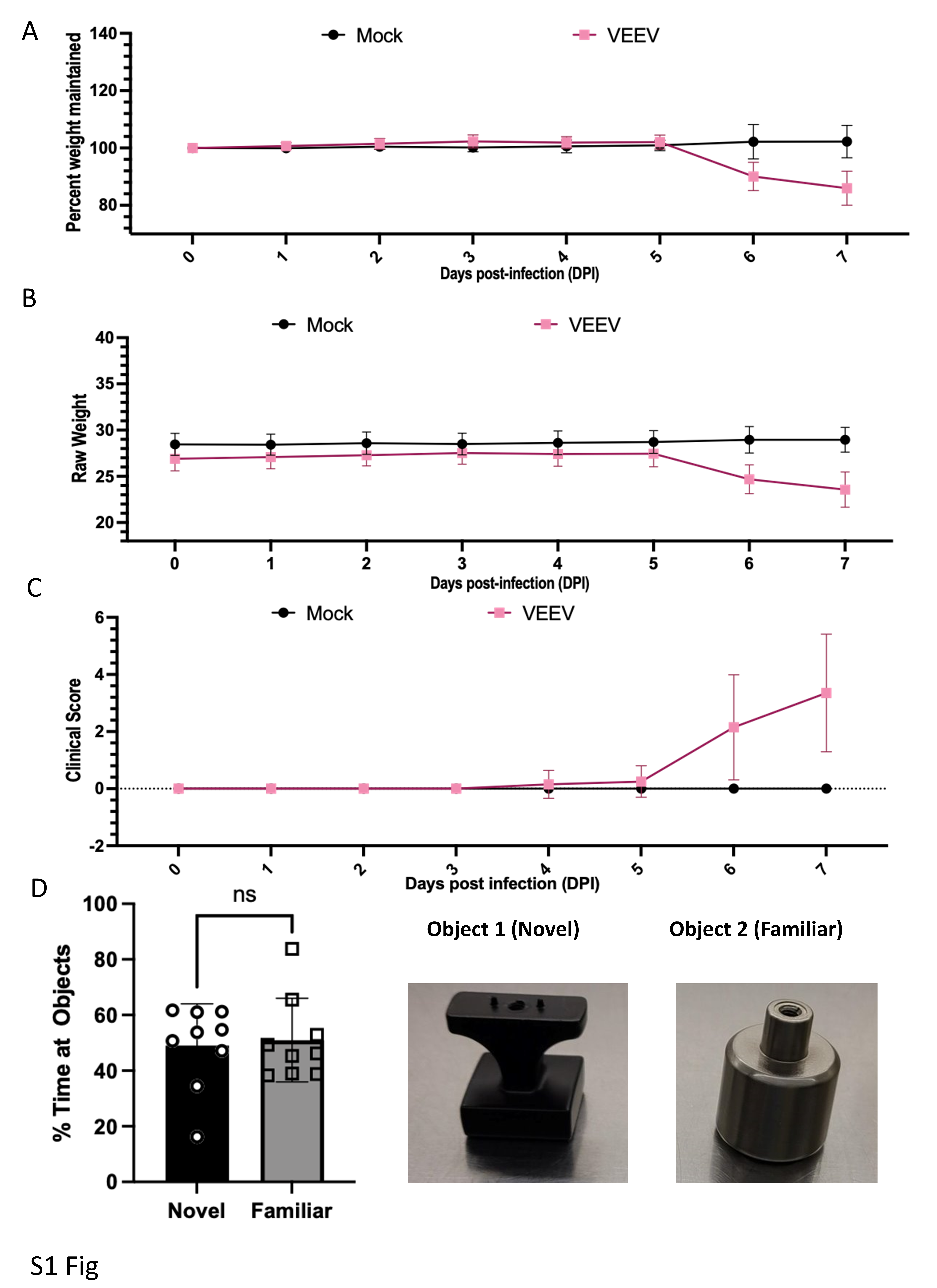

Supplement: S1 Fig — A) percent weight maintained, B) Raw weight (g), and C) Clinical score of 7 DPI cohort Mock vs VEEV-infected animals. D) Percent time at object 1 vs object 2 in naïve untrained mice. (TIF) [file ppat.1014115.s001.tif]

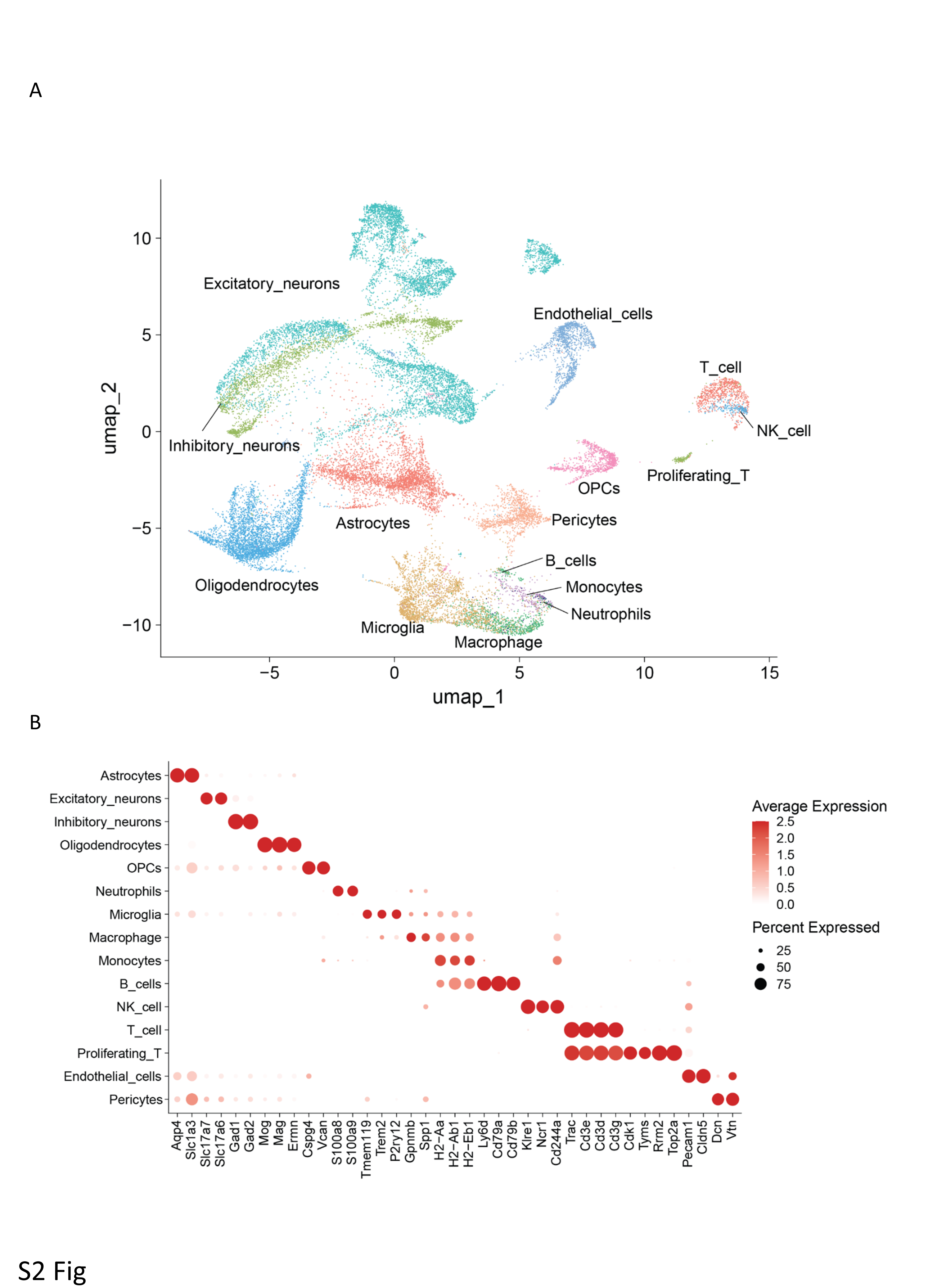

Supplement: S2 Fig — A) Heatmap map showing the expression level of selected cell type markers for each cluster. B) Dot plot showing the expression of select cell-type markers. (TIF) [file ppat.1014115.s002.tif]

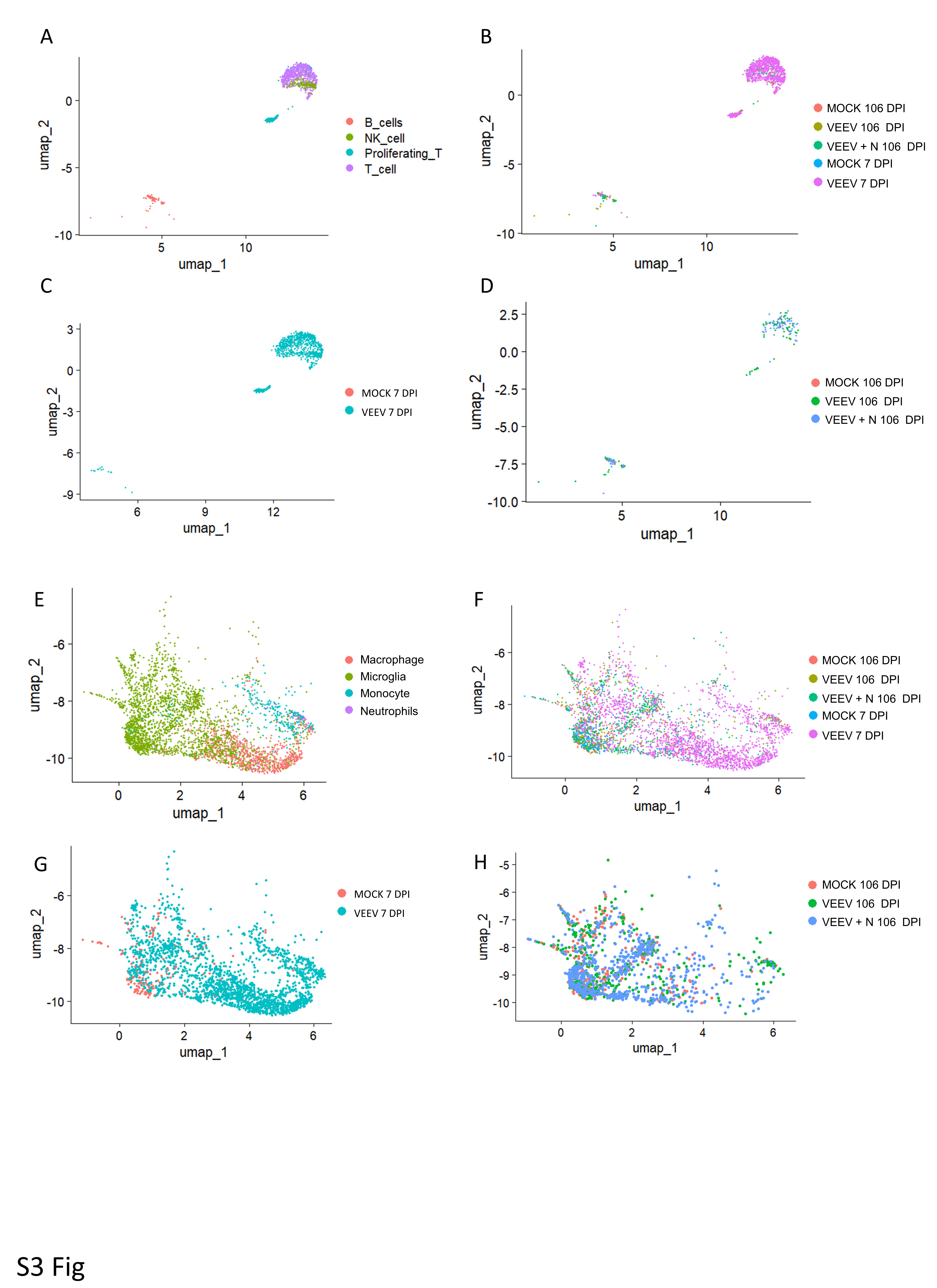

Supplement: S3 Fig — A) Total cell clusters identified as lymphoid immune cell clusters. B) Cell clusters containing B cells (red); Natural Killer (NK; green), Proliferating T-cells (blue), and T-cells (purple). C and D) lymphoid immune clusters colored by sample. Separated immune cell clusters for C) 7 DPI and D) 106 DPI. E) Total cell clusters identified as myeloid immune cell clusters. F) Cell clusters containing Macrophage (red); Microglia (green), Monocyte (blue), and Neutrophils (purple). G and H) Myeloid immune clusters colored by sample. Separated immune cell clusters for G) 7 DPI and H) 106 DPI. (TIF) [file ppat.1014115.s003.tif]

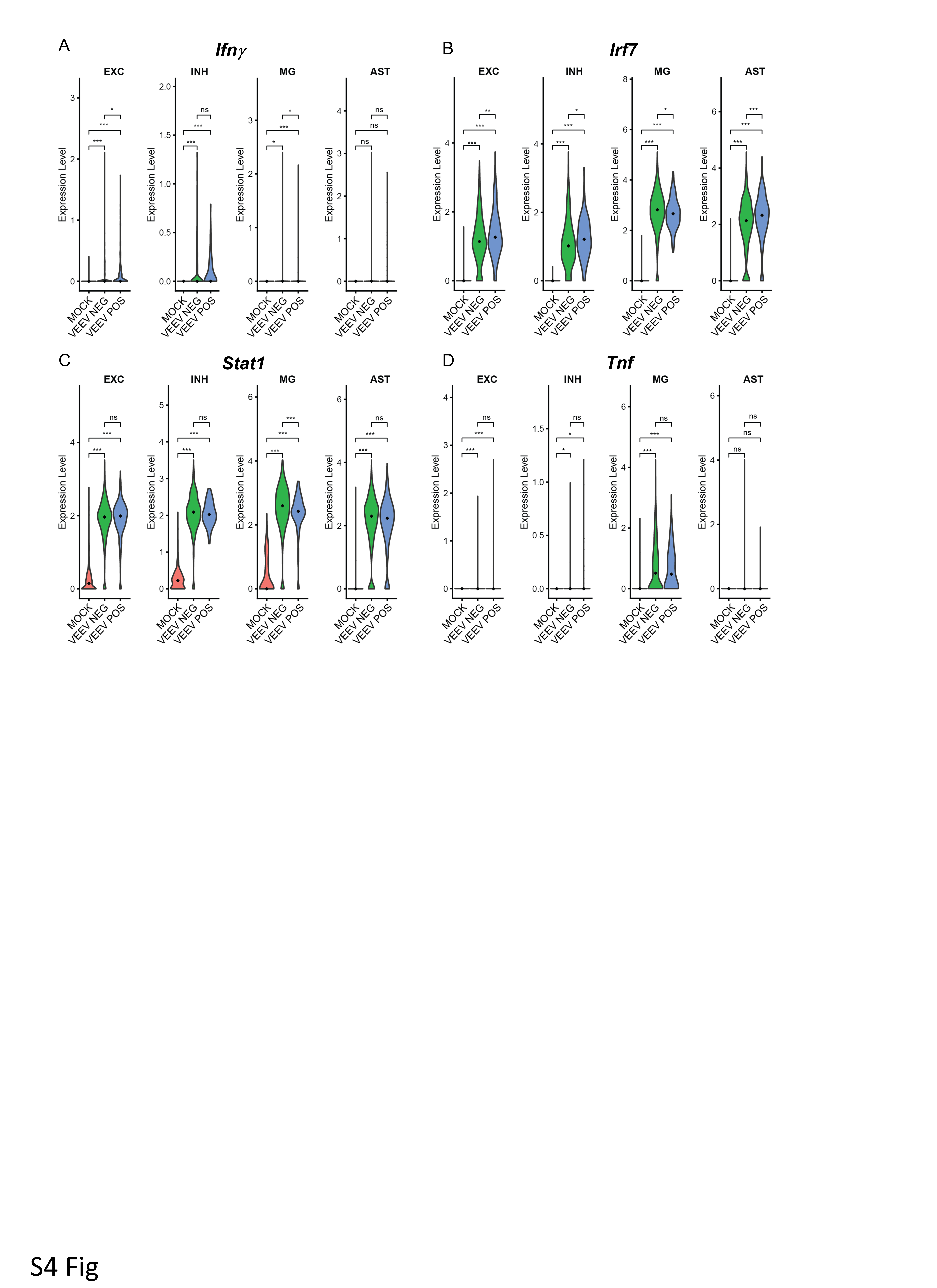

Supplement: S4 Fig — A) Ifnγ, B) Irf7, C) Stat1, and D) Tnf. Statistical significance was determined by Fisher’s exact test,* = p-value≤0.05,*** = p-value≤0.001,**** = p-value≤0.0001. (TIF) [file ppat.1014115.s004.tif]

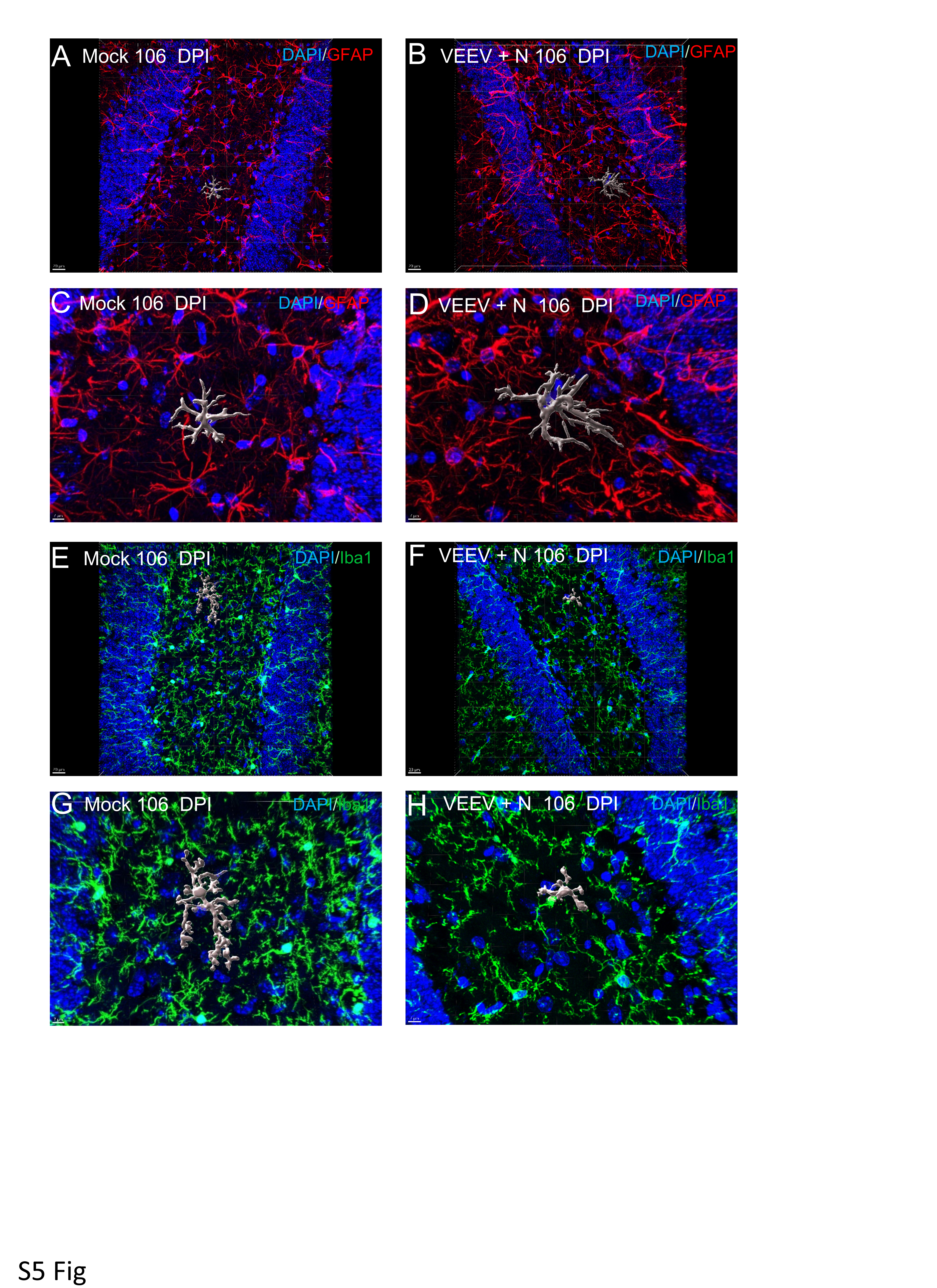

Supplement: S5 Fig — (A-B) GFAP+ cell 3D reconstruction in the hippocampus of Mock 106 DPI and VEEV + N 106 DPI mice, respectively. (C-D) Bottom panels show inset which reveals larger more hypertrophic astrocytes in VEEV + N mice. (E-F) Iba1 + cell 3D reconstruction in the hippocampus of Mock 106 DPI and VEEV + N 106 DPI mice, respectively. (F-H) Bottom panels show inset which reveals VEEV + N mice have a morphological shift in microglia morphology to ameboid shape. (TIF) [file ppat.1014115.s005.tif]

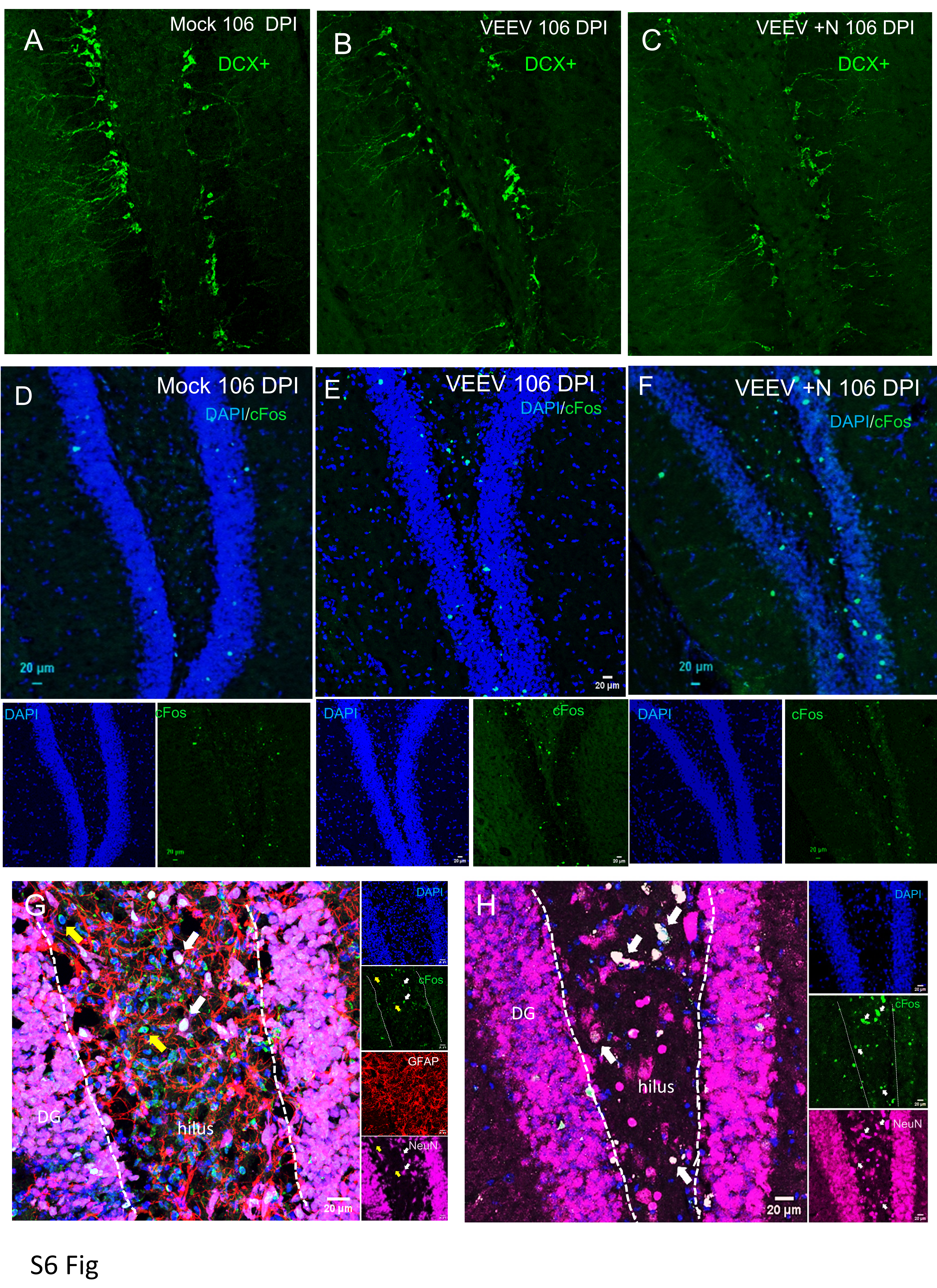

Supplement: S6 Fig — Doublecortin (DCX+) cells in A) Mock, B) VEEV, and C) VEEV + N at 106 DPI. cFos+ cells in A) Mock, B) VEEV, and C) VEEV + N at 106 DPI. G) Representative images of c-Fos expression overlayed with astrocytes (GFAP), and neurons (NeuN) in VEEV-infected animals at 7 DPI indicate partial overlap with neurons (NeuN; pink) (overlap indicated by white arrows, non-overlap indicated by yellow arrows) compared to astrocytes (GFAP; red) at 7 DPI, whereas H) 106 DPI displays c-Fos almost exclusively in neurons (white arrows). (TIF) [file ppat.1014115.s006.tif]
